# Genetic Evidence Reveals the Indispensable Role of the *rseC* Gene for Autotrophy and the Importance of a Functional Electron Balance for Nitrate Reduction in *Clostridium ljungdahlii*

**DOI:** 10.3389/fmicb.2022.887578

**Published:** 2022-05-09

**Authors:** Christian-Marco Klask, Benedikt Jäger, Isabella Casini, Largus T. Angenent, Bastian Molitor

**Affiliations:** ^1^Environmental Biotechnology Group, Geo- and Environmental Science Center, University of Tübingen, Tübingen, Germany; ^2^Cluster of Excellence – Controlling Microbes to Fight Infections, University of Tübingen, Tübingen, Germany; ^3^Max Planck Institute for Biology Tübingen, Tübingen, Germany

**Keywords:** acetogenic bacteria, *Clostridium ljungdahlii*, CRISPR, RNF-gene cluster, nitrate reduction, autotrophy

## Abstract

For *Clostridium ljungdahlii*, the RNF complex plays a key role for energy conversion from gaseous substrates such as hydrogen and carbon dioxide. In a previous study, a disruption of RNF-complex genes led to the loss of autotrophy, while heterotrophy was still possible *via* glycolysis. Furthermore, it was shown that the energy limitation during autotrophy could be lifted by nitrate supplementation, which resulted in an elevated cellular growth and ATP yield. Here, we used CRISPR-Cas12a to delete: **(1)** the RNF complex-encoding gene cluster *rnfCDGEAB*; **(2)** the putative RNF regulator gene *rseC*; and **(3)** a gene cluster that encodes for a putative nitrate reductase. The deletion of either *rnfCDGEAB* or *rseC* resulted in a complete loss of autotrophy, which could be restored by plasmid-based complementation of the deleted genes. We observed a transcriptional repression of the RNF-gene cluster in the *rseC*-deletion strain during autotrophy and investigated the distribution of the *rseC* gene among acetogenic bacteria. To examine nitrate reduction and its connection to the RNF complex, we compared autotrophic and heterotrophic growth of our three deletion strains with either ammonium or nitrate. The *rnfCDGEAB*- and *rseC*-deletion strains failed to reduce nitrate as a metabolic activity in non-growing cultures during autotrophy but not during heterotrophy. In contrast, the nitrate reductase deletion strain was able to grow in all tested conditions but lost the ability to reduce nitrate. Our findings highlight the important role of the *rseC* gene for autotrophy, and in addition, contribute to understand the connection of nitrate reduction to energy metabolism.

## Introduction

Acetogenic bacteria (i.e., acetogens), such as *Clostridium ljungdahlii*, maintain autotrophic growth with mixtures of the gaseous substrates carbon dioxide, carbon monoxide, and hydrogen as carbon and energy sources (Drake et al., 2008; Katsyv and Müller, 2020). The pathway that allows carbon fixation for autotrophic growth in acetogens is the Wood-Ljungdahl pathway (Wood et al., 1986; Ljungdahl, 2009). Overall, the Wood-Ljungdahl pathway is considered the most energy-efficient pathway for carbon fixation that exists in nature (Fast and Papoutsakis, 2012; Song et al., 2020). In the Wood-Ljungdahl pathway, two molecules of carbon dioxide are reduced to one carbonyl group and one methyl group, which are then combined with coenzyme A to the central metabolite acetyl-coenzyme A (Ljungdhal, 1986). The electrons for these reductions can be derived from the oxidation of hydrogen or carbon monoxide, while carbon monoxide can also enter the pathway directly to provide the carbonyl group (Wood, 1991). For carbon fixation, acetyl-coenzyme A is channeled into the anabolism for cellular growth (Ragsdale and Pierce, 2008). For energy conservation, acetyl-coenzyme A is converted to acetate, which generates cellular energy by substrate level phosphorylation (Schuchmann and Müller, 2014). One mole of ATP is generated per mole of acetate that is produced. However, in the first step of the pathway, after carbon dioxide was reduced to formate, one mole of ATP is invested to activate the formate to formyl-tetrahydrofolate (Wood et al., 1986; Ljungdahl, 2009). Thus, the energy balance of the Wood-Ljungdahl pathway alone is net zero (Schuchmann and Müller, 2014). All required cellular energy for the anabolism of the microbes during autotrophy is generated *via* membrane-coupled phosphorylation (Katsyv and Müller, 2020). In *C. ljungdahlii*, the membrane-bound transhydrogenase *Rhodobacter* nitrogen fixation (RNF) complex (Schmehl et al., 1993; Biegel et al., 2011) utilizes two electrons from the oxidation of reduced ferredoxin to reduce NAD^+^ to NADH, while simultaneously one proton is translocated across the membrane (Tremblay et al., 2012; Schuchmann and Müller, 2014). A proton-dependent F_1_F_O_ ATPase then consumes the chemiosmotic proton gradient to generate ATP (Köpke et al., 2010; Al-Bassam et al., 2018). In the presence of carbon dioxide and hydrogen, theoretically, *C. ljungdahlii* can generate a maximum of 0.63 moles ATP per mole acetate for the anabolism *via* membrane-coupled phosphorylation. Thus, the conservation of cellular energy during autotrophy occurs at the thermodynamic limit of life (Schuchmann and Müller, 2014).

For *C. ljungdahlii*, the RNF complex is encoded by the RNF-gene cluster *rnfCDGEAB*. Although the RNF complex plays an essential role for energy conservation during autotrophy in *C. ljungdahlii* (Tremblay et al., 2012), fundamental knowledge about the regulation and gene expression control of the encoding RNF-gene cluster is missing. Transcriptome studies with *C. ljungdahlii* revealed that the RNF complex is under strict gene expression control and strongly induced during autotrophy (Tan et al., 2013; Al-Bassam et al., 2018). The regulatory mechanisms behind this remain unknown. However, the small gene *rseC*, which is located directly upstream of *rnfC* in *C. ljungdahlii*, is also highly expressed during autotrophy and follows the expression profile of *rnfC* (Al-Bassam et al., 2018). The gene *rseC* is annotated to contain the conserved protein domain family RseC_MucC (pfam04246) (Köpke et al., 2010). The domain family RseC_MucC is found in positive transcriptional regulators in other microbes. The one representative, RseC, was found to be involved in the oxidative stress response in *Escherichia coli* (De Las Peñas et al., 1997; Missiakas et al., 1997; Koo et al., 2003), and in thiamine synthesis in *Salmonella Typhimurium* (Beck et al., 1997). The other representative, MucC, was found to be involved in the regulation of the alginate formation of *Azotobacter vinelandii* (Martinez-Salazar et al., 1996) and *Pseudomonas aeruginosa* (Boucher et al., 1997). Others identified a transcription start site for *C. ljungdahlii*, which is located upstream of the *rseC* gene, and a putative terminator sequence, which is located between *rseC* and *rnfC*. This indicates that *rseC* is expressed as an individual transcript apart from the RNF-gene cluster transcripts (Al-Bassam et al., 2018). Altogether, this led to the assumption that the *rseC* gene product is closely linked to the RNF complex, and could be important for the regulation of autotrophy in *C. ljungdahlii* (Köpke et al., 2010; Al-Bassam et al., 2018).

While autotrophy in acetogens results in low cellular energy yields, Emerson et al. (2019) reported that *C. ljungdahlii* is able to couple the reduction of nitrate to the generation of ATP during growth with carbon dioxide and hydrogen. This relieved the energy limitation during autotrophy and resulted in a significantly higher biomass yield (Emerson et al., 2019). We confirmed this in a bioreactor study, and biomass yields were considerably higher with nitrate, but resulted in stochastic crashes of the continuous bioreactor cultures (Klask et al., 2020). Emerson et al. (2019) proposed that electrons, which are required for nitrate reduction, are provided by NADH. One route to regenerate NADH is by the RNF complex where reduced ferredoxin is consumed (Biegel et al., 2011), which would link nitrate reduction to the energy metabolism. It was assumed that nitrate reduction is accelerating the RNF-complex activity, and thus the generation of ATP (Emerson et al., 2019). This way, the co-utilization of carbon dioxide and nitrate with hydrogen was suggested to yield up to 1.5 ATP through the concerted action of the RNF complex and the ATPase (Emerson et al., 2019). This would be a 2.4-fold increase in ATP yield compared to the ATP yield with carbon dioxide and hydrogen alone (Schuchmann and Müller, 2014).

To investigate the autotrophy in *C. ljungdahlii* with respect to regulatory aspects and the interplay with nitrate reduction, we addressed three main questions: (**1)** Is the *rseC* gene involved in the regulation of the RNF-gene cluster?; (**2)** Is nitrate reduction dependent on a functional RNF complex?; and (**3)** Is nitrate reduction abolished by the deletion of the nitrate reductase that is annotated in the genome of *C. ljungdahlii*?

## Materials and Methods

### Bacterial Strains and Growth

*Escherichia coli* TOP10 (Thermo Fisher Scientific, Massachusetts, United States), *E. coli* EPI300 (Lucigen, Wisconsin, United States), and *E. coli* HB101 pKR2013 (DSM 5599) were grown at 37°C in Luria Broth (LB) medium containing (per liter): 5 g NaCl; 10 g peptone; and 5 g yeast extract. *C. ljungdahlii* ATCC13528 was generally cultivated in anaerobic Rich Clostridial Medium (RCM) containing per liter: 5 g fructose; 3 g yeast extract; 10 g meat extract; 10 g peptone; 5 g NaCl; 1 g soluble starch; 3 g sodium acetate; 0.5 g L-cysteine HCl; and 4 mL resazurin-solution (0.025 vol-%). For growth experiments with *C. ljungdahlii*, standard PETC medium (Klask et al., 2020) was used containing (per liter): 1 g yeast extract; 1.0 g NH_4_Cl; 0.1 g KCl; 0.2 g MgSO_4_x7 H_2_O; 0.8 g NaCl; 0.1 g KH_2_PO_4_; 0.02 g CaCl_2_x2 H_2_O; 4 mL resazurin-solution (0.025 vol-%); 10 mL trace element solution (TE, 100x); 10 mL Wolfe’s vitamin solution (100x); 10 mL reducing agent (100x); and 20 mL of fructose/2-(N-morpholino)ethanesulfonic acid (MES) solution (50x). TE was prepared as 100x stock solution containing (per liter): 2 g nitrilotriacetic acid (NTA); 1 g MnSO_4_xH_2_O; 0.8 g Fe(SO_4_)2(NH_4_Cl)2 × 6 H_2_O; 0.2 g CoCl_2_x6 H2O; 0.0002 g ZnSO_4_x7 H_2_O; 0.2 g CuCl_2_x2 H_2_O; 0.02 g NiCl_2_x6 H_2_O; 0.02 g Na_2_MoO_4_x2 H_2_O; 0.02 g Na_2_SeO_4_; and 0.02 g Na_2_WO_4_. The pH of the TE was adjusted to 6.0 after adding NTA. The solution was autoclaved and stored at 4°C. Wolfe’s vitamin solution was prepared aerobically containing (per liter): 2 mg biotin; 2 mg folic acid; 10 mg pyridoxine-hydrochloride; 5 mg thiamin-HCl; 5 mg riboflavin; 5 mg nicotinic acid; 5 mg calcium pantothenate; 5 mg p-aminobenzoic acid; 5 mg lipoic acid; and 0.1 mg cobalamin. The vitamin solution was sterilized using a sterile filter (0.2 μm), sparged with N_2_ through a sterile filter, and stored at 4°C. The 50x fructose/MES solution contained (per 100 mL): 25 g fructose; and 10 g MES. The pH was adjusted to 6.0 by adding KOH. For autotrophic experiments, fructose was omitted. In nitrate experiments, ammonium chloride was replaced with sodium nitrate (NaNO_3_) in the equal molar amount (=18.7 mM). The reducing agent solution contained (per 100 mL): 0.9 g NaCl and 4 g L-cysteine HCl and was prepared with anaerobic water under anaerobic conditions. The reducing agent was stored at room temperature. For solid LB medium, 1.5 weight-% agar was added. For solid RCM or PETC medium 1.0–2.0 weight-% agar was added. For conjugation of *C. ljungdahlii* cells (see below) a modified PETC medium (PETC + 5gS) was used containing additionally (per liter): 5 g peptone and 5 g meat extract.

Liquid *E. coli* cultures and autotrophic *C. ljungdahlii* cultures were agitated at 150 revolutions per minute (rpm) (Lab Companion Incubater Shaker ISS-7100R, Jeio Tech, Daejeon, Republic of Korea). Heterotrophic cultures of *C. ljungdahlii* and LB plates with *E. coli* cells were incubated without shaking (Incubator IN260, Memmert, Schwabach, Germany). Anaerobic work was performed in an anaerobic chamber (Glovebox-System UNIlab Pro, MBraun, Garching bei München, Germany) with an N_2_ (100 vol-%) atmosphere. However, *C. ljungdahlii* cultures in bottles were transferred at the bench with sterile syringes and needles. Before each transfer between serum bottles, we flamed the top of the rubber stopper with ethanol (70 vol-%) at a Bunsen burner. All plating work with *C. ljungdahlii* was performed in the anaerobic chamber with a maximum of 5 parts per million (ppm) oxygen in the atmosphere. All plating work with *E. coli* was carried out in a laminar flow bench (Hera Safe KS18, Thermo Fischer Scientific, Massachusetts, United States). Antibiotics (see below) were added to maintain plasmid stability in recombinant cultures of *E. coli* and *C. ljungdahlii*.

### Antibiotics

Chloramphenicol (30 μg/mL), ampicillin (100 μg/mL), kanamycin (50 μg/mL), and trimethoprim (10 μg/ml) were applied to maintain plasmids in *E. coli* strains, while thiamphenicol (5 mg/mL) was used for recombinant strains of *C. ljungdahlii*. Trimethoprim was dissolved in DMSO (100 vol-%). Thiamphenicol was prepared as aerobic stock solution (25 mg/mL) in DMSO (100 vol-%) and diluted with sterile water (1:10) before use. The diluted thiamphenicol solution (2.5 mg/mL) was transferred into a sterile 1 mL syringe. 100 μL of this solution was used to add to a 50 mL RCM or PETC medium (final concentration of 5 μg/mL). The use of DMSO over ethanol as solvent for thiamphenicol prevented the addition of external ethanol, which is a metabolite, to cultures of *C. ljungdahlii*. All antibiotic stock solutions were stored at −20°C.

### General Cloning and Gene Manipulation

The broad-host shuttle-vector system pMTL80000 (Heap et al., 2009) was used for all cloning steps. All generated plasmids of this study (Supplementary Table 1) were cloned with restriction endonucleases and T4 ligase (New England Biolabs, Frankfurt am Main, Germany) or Gibson assembly (NEBuilder^®^ HiFi DNA Assembly, New England Biolabs, Frankfurt am Main, Germany). PCR work was carried out with primers provided by IDT (Integrated DNA Technologies, Iowa, United States) (Supplementary Table 2) and with a proof-reading Q5^®^ High-Fidelity DNA Polymerase (New England Biolabs, Frankfurt am Main, Germany)) according to the manufacturer’s guidelines. Genomic DNA (gDNA) was purified from 2 mL of exponential cultures of *C. ljungdahlii* with the NucleoSpin Tissue Mini kit (Macherey-Nagel, Düren, Germany) and used as PCR-template. Notably, instead of performing harsh cell disruption according to the manufacturer’s recommendation, we applied a 6 × 10 s vortex interval during the procedure. All PCR steps were performed with Q5^®^ High-Fidelity DNA Polymerase (New England Biolabs, Frankfurt am Main, Germany) and primers provided by IDT (Integrated DNA Technologies, Iowa, United States) (Supplementary Table 2). PCR products were purified with QIAquick PCR Purification Kit (Qiagen, Hilden, Germany).

#### Design and Generation of Clustered Regularly Interspaced Short Palindromic Repeats-FnCas12a Plasmids for Gene Deletion

The broad-host plasmid pMTL83152 (Heap et al., 2009) was used as backbone (Supplementary Table 1). The gene *Fncas12a* of *Francisella novicida* (Zetsche et al., 2015) was obtained from plasmid pY001 (Addgene #69973) and amplified with primers cas12a_fwd_*Bam*HI and cas12a_rv_*Nco*I generating *Bam*HI and *Nco*I restriction sites for a subsequent restriction cloning to generate pMTL83152_*Fn*Cas12a. Two homology-directed repair arms (HDR1/HDR2) each with a size of 1,000–1,200 bp, which flank the targeted gene, were individually amplified with HDR_upst_fwd/rv and HDR_dwst_fwd/rv primers generating an overlap of 25–40 bp to each other. The fragments were purified, and 50–100 ng of both fragments were used as template for a subsequent fusion PCR using HDR_upst_fwdOv and HDR_dwst_rvOv primers, which generated new overlaps at 5′ and 3′ (fusion fragment HDR1/2). An crRNA array was synthesized and cloned as minigene into plasmid pUC19 by IDT (Integrated DNA Technologies, Iowa, United States) (Supplementary Table 3). The crRNA array sequence contained the mini-promoter P4 (5′-TTGACAAATTTATTTTTTAAAGTTAAAATTAAGTTG-3′) (Xu et al., 2015), the FnCas12a-specific directed repeats (DR) sequence (5′-TAATTTCTACTGTTGTAGAT-3′) (Zetsche et al., 2015), 1–2 sgRNA for the targeted gene(s) (Pam sequence TTV for target RNF and TTTV for target *rseC* and *nar*), and the rrbn-T1-terminator (Orosz et al., 1991). The crRNA array fragment was amplified with primers minigene_crRNA_fwd/rv creating overhangs to the fused HDR1/2 fragment and the plasmid backbone. For gene targets with a size > 2 kb, such as *rnfCDGEAB* and *nar*, two sgRNA (and two DRs) were used in the same crRNA array (Supplementary Table 3). For the assembly reaction (Gibson Assembly Ultra Kit, Synthetic Genomics, California, United States), the plasmid pMTL83152_*Fncas12a* was first digested using *Bbv*CI and CIP (New England Biolabs, Frankfurt am Main, Germany) for 3 h at 37°C, purified by PCR-clean, and then mixed with the purified fused HDR1/2 fragment and the crRNA array fragment. Using electrocompetent *E. coli* EPI300 cells (TransforMax™, Lucigen, Wisconsin, United States) and electroporation for transformation highly increased cloning efficiency for the CRISPR-Cas12a constructs in *E. coli*. For inducible Cas12a expression, the P*_*thl*_* module was replaced with the *tetR-O1* promoter module (P*_*tetR*–*O*1_*) (Woolston et al., 2018) using restriction sites *Sbf*I and *Bam*HI, for all generated CRISPR-Cas12a plasmids.

#### Generation of Overexpression and Complementation Plasmids

The *rnfCDGEAB* gene cluster (CLJU_c11360-410) and a 213-bp sequence located upstream of *rnfC*, which contains the putative native promoter sequence (P_nat_), were amplified as one fragment using primers rnfCDGEAB + 213 bp_fwd and rnfCDGEAB_rv. The *rseC* gene (CLJU_c11350) was amplified using primers *rseC*_fwd and *rseC*_rv. The gene cluster CLJU_c23710-30, here referred to as *nar*, was amplified as one fragment using primers nar-full_fwd and nar-full_rv. All PCR products were purified with the QIAquick PCR Purification kit (Qiagen, Hilden, Germany). Subsequently, the purified fragments were ligated into pMinit2.0 (New England Biolabs, Frankfurt am Main, Germany) and used for transformation of CaCl_2_-competent *E. coli* TOP10 cells (Sambrook and Russell, 2006). Next, the plasmid DNA was digested using the restriction sites determined by the used PCR primers and the fragment was cloned into the pMTL83151 plasmid generating pMTL83151_P_nat__*rnfCDGEAB* or into the pMTL83152 plasmid generating pMTL83152_*rseC* and pMTL83152_*nar*. Subsequently, all cloned fragments were verified again with test-digestion of the plasmid DNA and Sanger sequencing to exclude mutations in the gene sequences.

### Screening for Correct Plasmid DNA and Genome Editing

For screening and continuous purity control of our *C. ljungdahlii* strains (Supplementary Table 4), we performed PCRs from culture samples or from purified DNA with the Phire Plant Direct PCR Master Mix (Thermo Fischer Scientific, Massachusetts, United States). *E. coli* colonies grown on selective LB plates after receiving plasmid constructs were analyzed for the correctly assembled plasmids using the Phire Plant Direct PCR Master Mix (Thermo Fischer Scientific, Massachusetts, United States). A small amount of recombinant *E. coli* cell material was directly transferred to the reaction mix. For *C. ljungdahlii* cells, 0.5–1 mL culture sample was harvested by centrifugation for 3 min at 13,806 rpm (Centrifuge 5,424, FA-45-24-11, Eppendorf, Hamburg, Germany) and resuspended in 100–500 μL 10 mM NaOH depending on the size of the cell pellet. Subsequently, cell suspensions were boiled for 10 min at 98°C. The hot reaction tubes were incubated on ice for 1 min and quickly vortexed before they served as a DNA template. In general, we used 20 μL PCR master mix, which consisted of 10 μL (Thermo Fisher Scientific, Massachusetts, United States) 2x, 0.8 μL of each primer, 1 μL cell lysate sample, and 7.4 μL nuclease-free water. The PCR reaction was carried out according to the manufacturer’s guidelines. We generally used the primers tra60 bp_fwd and repH_401 bp_rv or repH_643 bp_rv for these control PCRs (Supplementary Table 2), because they bind to the plasmid backbone of every pMTL plasmid used in this study. Verification of gene deletion in the genome of *C. ljungdahlii* was performed with “outside” primers, which bound upstream and downstream of the used homology-directed repair arms (HDR1/2) on the genomic DNA (Supplementary Table 2). In addition, we performed test-digestion of the generated plasmids with restriction enzymes (New England Biolabs, Frankfurt am Main, Germany), and analyzed the fragment pattern *via* gel electrophoresis. The final plasmid sequence was verified by Sanger sequencing. Plasmid DNA was purified from *E. coli* with self-made purification buffers (described below). Correct plasmid DNA was then purified with the QIAprep Spin Miniprep kit (Qiagen, Hilden, Germany) *prior* to further use.

### A Fast Method for Plasmid Purification From *Escherichia coli* Without Use of a Commercial Kit

For screening of successfully transformed *E. coli* cells, we used a time- and money-saving protocol to purify plasmid DNA from multiple samples without using a commercial kit, which is a modified alkaline lysis protocol adapted from Sambrook et al. (1989). All centrifugation steps were performed at 13,806 rpm for 5 min (Centrifuge 5,424, FA-45-24-11, Eppendorf, Hamburg, Germany). Recombinant *E. coli* cells were grown overnight in 5 mL selective liquid LB at 37°C and 150 rpm. 1.5–3 mL cell suspension were harvested in 1.5 mL reaction tubes. The supernatant was discarded, and the pellet was resuspended by vortexing in 150 μL P1-buffer (50 mM Tris, 10 mM EDTA, 100 μg/mL RNAseA, pH 8.0 with HCl). Cells were lysed in 150 μL P2-buffer (200 mM NaOH, 1 vol-% SDS) and inverted five times. Proteins were precipitated by adding 250 μL P3-buffer (2.55 M Na-acetate, pH 4.8 with acetic acid). The samples were inverted five times and centrifuged. Subsequently, 500 μL of the supernatant were transferred into new 1.5 mL tubes and mixed with 500 μL isopropanol. The samples were quickly vortexed and centrifuged again. Afterward, the supernatant was discarded. At this step, the precipitated and non-visible plasmid-DNA pellet remained on the bottom of the tube. The pellet was washed twice with ice-cold ethanol (70 vol-%) omitting resuspending the DNA. After the second washing, the supernatant was discarded completely and the remaining ethanol was first removed by snapping the tube on a piece of clean paper towel and then through drying at 50–65°C for 10 min. The dried pellet was resuspended in 30 μL elution buffer (Tris/EDTA, pH 7.2) or deionized water. Purified plasmid-DNA with a concentration of 250–500 ng/μL was clean enough for subsequent cloning steps and test-digestion, however, an additional clean-up with the QIAquick PCR Purification Kit (Qiagen, Hilden, Germany) was carried out when a Sanger sequencing reaction was necessary. P1-buffer needed to be stored at 4°C to maintain RNAse activity for up to 3 months. P2- and P3-buffer were stored at room temperature.

### A Modified Conjugation Protocol for *Clostridium ljungdahlii*

This protocol was adapted and modified according to Mock et al. (2015). Cells of *C. ljungdahlii* were grown in RCM overnight to mid exponential growth until an OD_600_ of 0.4–0.8 was reached (NanoPhotometer^®^ NP80, Implen, München, Germany). *E. coli* HB101 pKR2013 (DSM 5599) harboring the desired CRISPR-Cas12a-plasmid was grown as pre-culture in selective 5 mL LB medium overnight. The plasmid pKR2013 contains essential genes to mediate conjugation and a kanamycin resistance cassette. 1–2 mL of the *E. coli* cells were used to inoculate 10 mL selective LB medium in 50 mL baffled flask and cultivated until mid-exponential growth (OD_600_ 0.5–1.0). Subsequently, the *E. coli* culture was cooled to 4°C and 2 mL were transferred into sterile 2 mL reaction tubes. The *C. ljungdahlii* culture was kept at room temperature until use. Inside the anaerobic chamber, *E. coli* cells were centrifuged softly at 2,900 rpm (mySpin™ 12 mini centrifuge, Thermo Fischer Scientific, Massachusetts, United States) to protect pili, and washed once with sterile and anaerobic 0.1 M phosphate buffered saline (PBS) at pH 6.0. Afterward, the washed pellet was resuspended gently in 100–150 μL cell suspension of *C. ljungdahlii* and directly transferred to well-dried RCM-agar plates (2 vol-% agar). Spot-mating was carried out at 37°C inside the anaerobic chamber overnight. After 8–24 h the spot was resuspended with anaerobic PBS (pH 6.0) and centrifuged at 10,000 rpm (mySpin™ 12 mini centrifuge, Thermo Fischer Scientific, Massachusetts, United States) for 3 min. The supernatant was discarded, and the pellet was resuspended in the remaining volume of the tube. Subsequently, 100 μL of the cell suspension was plated onto selective PETC + 5gS-agar plates, which contained 5 g/L of peptone and 5 g/L meat extract to support growth. Selective agar plates should not be older than 2–3 days. Thiamphenicol was added for plasmid selectivity. Trimethoprim (10 mg/mL) was added to counter-select against *E. coli*. Growth was obtained after 4–5 days at 37°C inside the anaerobic chamber. *C. ljungdahlii* colonies were transferred into (Glasgerätebau Ochs, Bovenden, Germany) containing 5 mL RCM with the respective antibiotics. A successful transformation of *C. ljungdahlii* with the correct plasmid was confirmed as follow: (**1**) growth in selective RCM with a characteristic pH decrease due to acetogenesis; (**2**) control PCRs with primers for plasmid specific fragments; and (**3**) plasmid purification from the culture and re-transformation into *E. coli* TOP10 cells.

### Electroporation of *Clostridium ljungdahlii* Cells

Electroporation of *C. ljungdahlii* cells was performed as previously reported (Xia et al., 2020) and applied for all non-CRISPR-based plasmids. Single colonies growing on selective plates were verified by PCR analyses and by re-transformation of *E. coli* with plasmid DNA, which was extracted from *C. ljungdahlii*.

### Growth Experiments With *Clostridium ljungdahlii*

In general, all recombinant *C. ljungdahlii* strains were pre-grown in 50 mL RCM in 100 mL serum bottles for 24–48 h. Subsequently, 2 mL cell suspension were used to inoculate 50 mL PETC medium in 100 mL (Glasgerätebau Ochs, Bovenden, Germany). This PETC pre-culture was cultivated for 40–48 h at 37°C until mid-exponential growth phase at OD_600_ of 0.5–1.0. Afterward, cells were transferred anaerobically into 50 mL reaction tubes, which were equilibrated for 3–5 days inside the anaerobic chamber. Cell harvest was performed outside the anaerobic chamber at 3,700 rpm for 12 min (Centrifuge 5920R, S-4 ×1,000, Eppendorf, Hamburg, Germany) at room temperature. After the centrifugation, the tubes were transferred back immediately into the anaerobic chamber, to keep the time at aerobic conditions at a minimum. Inside the anaerobic chamber, the supernatant was discarded, and the pellet was resuspended in fresh PETC medium to adjust to an OD_600_ of 5–10. The concentrated cell suspension was then transferred into sterile and anaerobic 10 mL (Glasgerätebau Ochs, Bovenden, Germany), sealed carefully, and used to inoculate main cultures outside of the anaerobic chamber. 1 mL of the cell suspension was used to inoculate 100 mL PETC main cultures. For heterotrophic growth experiments, 240 mL (Glasgerätebau Ochs, Bovenden, Germany) were used. Autotrophic growth experiments were performed in 1,000 mL Duran pressure plus bottles (Schott, Mainz, Germany), to provide a high medium-to-headspace ratio. The Duran pressure plus bottles were sealed with butyl stoppers and a GL45 ring cap. Before inoculation of autotrophic cultures, the N_2_ headspace was replaced with a sterile gas mixture consisting of H_2_/CO_2_ (80/20 vol-%). Each bottle contained 0.5 bar overpressure. All cultures were cultivated in biological triplicates as batch cultures. The gas headspace was not refilled during the experiments. However, for the strain *C. ljungdahlii* pMTL83151_P_nat__*rnfCDGEAB* and the control strain *C. ljungdahlii* pMTL83151 we refilled the headspace during this experiment with the same gas mixture to 0.5 bar overpressure at time points 44.5, 73.5, and 148.5 h (section “Plasmid-Based Complementation Relieved the Phenotypes of the *Clostridium ljungdahlii* ΔRNF and ΔrseC Strains”). We did not measure the headspace gas composition during our experiments. Culture samples of 3 mL were taken at the bench and used for: (**1**) OD_600_ measurement; (**2**) pH measurement; (**3**) HPLC analyses (acetate and ethanol); and (**4**) FIA analyses (nitrate, nitrite, and ammonium). All culture samples were stored at −20°C until use. OD_600_ samples were diluted with medium or PBS buffer when the absorbance was > 0.5. We applied a two-tailed Student’s *t*-test for all cultivation data. All *p-*values (*P*) below 0.001 indicate high significance and are given as ≤ 0.001.

### HPLC Analyses

HPLC analyzes were performed as described before (Klask et al., 2020). In addition, all frozen supernatant samples were thawed at 30°C for 10 min and 300 rpm, vortexed briefly, and centrifuged for 3 min at 13,806 rpm (Centrifuge 5,424, FA-45-24-11, Eppendorf, Hamburg, Germany) before use. All HPLC samples were randomized.

### Measurement of Nitrate, Nitrite, and Ammonium

Nitrate and nitrite concentrations were measured in a FIA continuous-flow analyzer system (AA3 HR AutoAnalyzer System, Seal Analytical GmbH, Norderstedt, Germany) as described before (Klueglein et al., 2014). Briefly, nitrate is reduced to nitrite with hydrazine and then reacts with sulfanilamide and NEDD (N-1-Naphthylethylenediamine di-HCl) to form a pink complex, which can be quantified photo-metrically at 550 nm. The protocol follows the DIN 38405/ISO 13395 standard methods. Ammonium concentrations were measured in the same system but with salicylate and dichloroisocyanuric acid forming a blue complex that is measured at 660 nm instead. The protocol was following DIN 38406/ISO 11732 standard methods. Culture samples of *C. ljungdahlii* were treated as explained above for HPLC preparation. However, we prepared 1:50 dilution in 1 mL with deionized water *prior* to the FIA analyses. Standards for nitrate, nitrite, and ammonium were measured before and during the analyses for a standard curve and to minimize drift effects. Nitrate concentrations of each sample were calculated by the difference of the amount of nitrite measured with and without the *prior* reduction by hydrazine.

### Growth Experiment for RNA Extraction From *Clostridium ljungdahlii*

For the expression analyses, we grew the strains *C. ljungdahlii* WT, *C. ljungdahlii* ΔRNF, and *C. ljungdahlii* Δ*rseC* under autotrophic and heterotrophic conditions as described above. The cultivation medium was PETC with ammonium as nitrogen source. Pre-cultures were grown in heterotrophic medium for 48 h. Next, the cells were transferred into the anaerobic chamber and harvested for 12 min at 25°C and 3,700 rpm (Centrifuge 5,920 R, S-4 × 1,000, Eppendorf, Hamburg, Germany) outside of the anaerobic chamber. The supernatant was discarded under anaerobic conditions and the pellet was resuspended in fresh medium of the main cultures. The start OD_600_ for autotrophic main cultures was 0.2, while it was 0.15 for heterotrophic conditions. The cultures were cultivated at 37°C. 10 mL culture samples were taken after 3 and 20 h. The samples were immediately cooled on ice and centrifuged for 12 min at 4°C and 3,700 rpm (Centrifuge 5,920 R, S-4 × 1,000, Eppendorf, Hamburg, Germany). The cell pellets were stored at −20°C until RNA extraction.

RNA was purified from *C. ljungdahlii* with the RNeasy Mini Kit (Qiagen, Hilden, Germany) as described before (Liu et al., 2013). For the RNA extraction, we used 2× 10^8^ cells, which was approximately 10 mL of a *C. ljungdahlii* culture at OD_600_ 0.2. The cell lysis was performed in the lysis buffer of the kit with 50 mg glass beads (0.1 mm silica spheres, MP Biomedicals, Eschwege, Germany, Eschwege, Germany) in a bead beater (5G FastPrep, MP Biomedicals, Eschwege, Germany) for 2 × 60 s at 9 m/s. RNA samples were eluted in 30 μL nuclease-free water. After the extraction procedure, an additional DNase I digest (RNase free Kit, Thermo Fisher Scientific, Massachusetts, United States) was performed to remove potential DNA contamination. Elimination of genomic DNA was confirmed with PCR analyses and gel electrophoresis. cDNA synthesis was performed with the QuantiTect Reverse Transcriptase Kit (Qiagen, Hilden, Germany) according to the manufacturer’s instructions. We used 500 ng RNA as template for each reaction. cDNA was stored at −20°C until further use.

### qRT-PCR Analyses

All qRT-PCR analyses were performed in a Quantstudio 3 Thermocycler (Applied Biosystems, Thermo Fisher Scientific, Massachusetts, United States). The PCR reaction mix contained 10 μL SYBR Green Master mix (Thermo Fisher Scientific, Massachusetts, United States), 1 μL of a fwd and rv qRT-PCR primer (final concentration 500 nM) (Supplementary Table 2), and 1 μL (∼5 ng) cDNA template. We used the *rho* gene as reference gene, which was described before as suitable candidate for qRT-PCR experiments with *C. ljungdahlii* (Liu et al., 2013). We added RNA controls to further exclude gDNA contamination in our samples. All qRT-PCR reactions were performed in technical triplicate according to the manufacturer’s instructions. We set the Ct threshold to 0.1. The fold change in gene expression between the samples was determined with the 2^–ΔΔCt^ method as described before (Livak and Schmittgen, 2001). We examined the PCR efficiency of our qPCR master mix by using plasmid DNA containing the sequence of each tested gene in a series of dilutions (10^–1^, 10^–2^, 5 × 10^–3^, 10^–3^, 5 × 10^–4^, 10^–4^). The slopes were ranging from 0.04 to 0.09 for the RNF-gene cluster genes and 0.17 for *rseC*, and were thus, close to zero, which proofs that the efficiencies are similar and the 2^–ΔΔCt^ can be used for interpretation of the qRT-PCR data (Livak and Schmittgen, 2001). We applied a two-tailed Student’s *t*-test based on our ΔCt values for each gene to analyze the significance of our samples in comparison to the wild type.

### Strain Preservation

Cultures of *C. ljungdahlii* were stored at −80°C. For this, cultures were grown in RCM until late exponential growth phase (OD_600_ 0.8–1.2) at 37°C for 36–48 h. The cells were transferred into anaerobic 50 mL reaction tubes inside the anaerobic chamber and harvested outside of the anaerobic chamber for 12 min at 4°C and 3,700 rpm (Centrifuge 5,920 R, S-4 × 1,000, Eppendorf, Hamburg, Germany). The supernatant was discarded inside the anaerobic chamber and the pellet was resuspended in fresh RCM medium to an OD_600_ of 5–10. Two milliliter of the cell suspension was transferred into 10 mL serum bottles, which were previously filled with 2 mL of 25–50 vol-% anaerobic and autoclaved glycerol. The serum bottles were briefly vortexed outside the anaerobic chamber, incubated on ice for 10–15 min and subsequently frozen at −80°C. For inoculation of a new RCM culture, a single serum bottle was quickly thawed up under rinsing water and 1–2 mL of the cell suspension was immediately transferred with a syringe into the medium bottle. Cultures of *E. coli* were stored at −80°C in sterile screw-cap tubes filled with 25–50 vol-% glycerol.

## Results

### A Full Deletion of the *Rhodobacter* Nitrogen Fixation Complex Confirmed Its Indispensable Role for Autotrophy in *Clostridium ljungdahlii*

**FIGURE 1:**
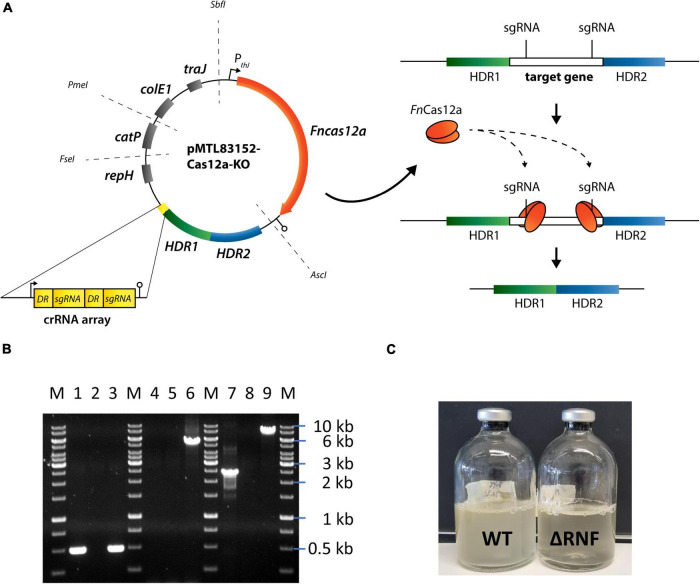
CRISPR-Cas12a-mediated *rnfCDGEAB* gene cluster deletion in *C*. *ljungdahlii*. **(A)** Modular CRISPR-Cas12a system established in the pMTL80000 shuttle-vector system (Heap et al., 2009). The final CRISPR-Cas12a plasmid for deletion of *rnfCDGEAB* contained the *Fncas12a* gene, homology-directed repair arms (HDRs), and a specific crRNA array comprising two directed repeats (DRs) and two sgRNA, which targeted the *rnfC* and *rnfB* genes. **(B)** Agarose gel with PCR-samples for the *fdhA* fragment (WT: 501 bp, deletion strain: 501 bp), *rnfCDGEAB* fragment (WT: 5,047 bp, deletion strain: no fragment), and for a fragment that was amplified with primers that bind ∼1,250 bp upstream and downstream of the *rnfCDGEAB* gene cluster locus (WT: 7,550 bp, deletion strain: 2,503 bp). DNA-template: gDNA of *C. ljungdahlii* ΔRNF (lane 1, 4, and 7); gDNA of *C. ljungdahlii* WT (lane 3, 6, and 9); and water (lane 2, 5, 8). M: Generuler™ 1 kb DNA ladder. **(C)** Growth of the wild type (WT) and reduced growth of the deletion strain (ΔRNF) with fructose in PETC medium. HDR1/2, homology-directed repair arm flanking the targeted gene; crRNA array, sequence containing FnCas12a-specific DRs and sgRNAs; sgRNA, guide RNA; *repH*, Gram-positive origin of replication; *catP*, antibiotic resistant cassette against chloramphenicol/thiamphenicol; *colE1*, Gram-negative origin of replication; *traJ*, conjugation gene; P*_*thl*_*, promoter sequence of the thiolase gene in *Clostridium acetobutylicum*; *Asc*I, *Fse*I, *Pme*I, and *Sbf*I are unique-cutting restriction sites, which were preserved during the cloning to maintain the modular functionality of the plasmid backbone.

**FIGURE 2:**
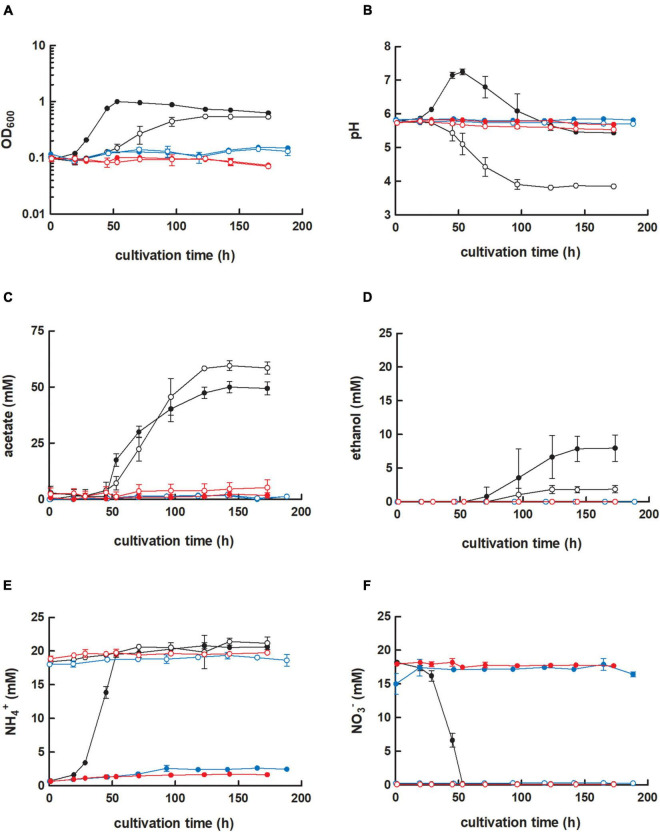
Cultivation of *C. ljungdahlii* WT, *C. ljungdahlii* ΔRNF, and *C. ljungdahlii* Δ*rseC* in nitrate- or ammonium-containing medium with H_2_ and CO_2_. Cultures of *C. ljungdahlii* strain WT (●, ◦), ΔRNF (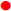, 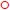), and Δ*rseC* (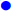, 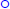) were grown in 100 mL PETC medium in 1 L bottles at 37°C and 150 rpm. The headspace consisted of H_2_ and CO_2_ (80/20 vol-%) and was set to 0.5 bar overpressure. The medium contained either 18.7 mM nitrate (NO_3_^–^) (filled circles) or 18.7 mM ammonium (NH_4_^+^) (open circles) as nitrogen source. The cultivation times were 173 h for cultures of *C. ljungdahlii* WT and *C. ljungdahlii* ΔRNF and 186 h for cultures of *C. ljungdahlii* Δ*rseC*. All cultures were grown in biological triplicates, data is given as mean values, with error bars indicating the standard deviation. **(A)** Growth; **(B)** pH-behavior; **(C)** acetate concentrations; **(D)** ethanol concentration; **(E)** ammonium concentration; and **(F)** nitrate concentrations. WT, wild type; ΔRNF, RNF-gene cluster deletion; Δ*rseC*, *rseC* gene deletion.

First, we achieved a full deletion of the RNF-gene cluster in *C. ljungdahlii* with a clustered regularly interspaced short palindromic repeats (CRISPR)-associated protein Cas12a (CRISPR-Cas12a) system, which we implemented and used to generate all deletion strains in this study (Figure 1A and Supplementary Text 1A). We confirmed the identity of the *C. ljungdahlii* ΔRNF strain (Figure 1B and Supplementary Text 1B), and compared the growth of *C. ljungdahlii* wild-type (WT) to the growth of *C. ljungdahlii* ΔRNF. We performed growth experiments with carbon dioxide and hydrogen (autotrophy) and with fructose (heterotrophy), while we added equimolar amounts of either ammonium or nitrate as nitrogen source to the medium for both autotrophy and heterotrophy (section “Materials and Methods,” Figure 2 and Supplementary Figure 1). As expected, we observed growth for *C. ljungdahlii* WT in all growth experiments (Figure 2A and Supplementary Figure 1A). However, the nitrogen source had a distinct influence on the growth rate, final OD_600_, fermentation product spectrum, and pH (Table 1, Figure 2B, Supplementary Text 1C, and Supplementary Figure 1B). We found that nitrate reduction occurred rapidly in our growth experiments (Figure 2F and Supplementary Figure 1F). *C. ljungdahlii* WT utilized all provided nitrate within 53 h of cultivation with carbon dioxide and hydrogen (Figure 2F) and within 47 h of cultivation with fructose (Supplementary Figure 1F). The ammonium concentrations increased concomitant with decreasing nitrate concentrations when nitrate was provided in the medium (Figure 2E and Supplementary Figure 1E). Noteworthy, we also observed an increase in the ammonium concentration when ammonium was provided as the nitrogen source during autotrophy (Figure 2F). We had added a small amount of yeast extract (0.1 weight-%) in all cultivation conditions (section “Materials and Methods”). We found this increase in the ammonium concentration in all our cultivation experiments, and we argued that the additional ammonium was a by-product of the fermentation of the added yeast extract in our cultivation medium (section “Materials and Methods,” Supplementary Text 1C). We did not measure any nitrite as an intermediate of the nitrate reduction pathway (discussed below).

**TABLE 1:** Performance of all tested *C. ljungdahlii* strains in autotrophic batch cultivation experiments.

Strain	Nitrogen source	Growth rate (μ in h^−1^)^a^	Maximum OD_600_ value	Maximum acetate concentration (mM)	Maximum ethanol concentration (mM)
WT	Ammonium	0.024 ± 0.002	0.56 ± 0.01	59.5 ± 1.8	1.9 ± 0.4
WT	Nitrate	0.072 ± 0.004	1.00 ± 0.06	50.1 ± 2.1	8.0 ± 1.6
ΔRNF	Ammonium	−	−	5.7 ± 3.0	n.d.^b^
ΔRNF	Nitrate	−	−	2.3 ± 1.1	n.d.^b^
Δ*rseC*	Ammonium	−	−	2.0 ± 0.5	n.d.^b^
Δ*rseC*	Nitrate	−	−	1.9 ± 0.1	n.d.^b^
Δ*nar*	Ammonium	0.018 ± 0.001 (−24%, *)	0.44 ± 0.01 (−21%,***)	44.8 ± 0.2 (−25%, ***)	3.3 ± 0.2 (+79%, *)
Δ*nar*	Nitrate	0.017 ± 0.003 (−76%, ***)	0.44 ± 0.01 (−55%,***)	41.9 ± 1.9 (16%,*)	2.9 ± 0.4 (−64%,*)

*Cultures were grown with carbon dioxide and hydrogen (autotrophy) in PETC medium, which contained either ammonium or nitrate as nitrogen source. A gas atmosphere of H_2_/CO_2_ (80/20 vol-%) with 0.5 bar overpressure was applied. Growth was not detected for any culture of C. ljungdahlii ΔRNF or C. ljungdahlii ΔrseC. Data is represented as mean values from biological triplicates ± standard deviation. WT, C. ljungdahlii wild type; ΔRNF, C. ljungdahlii with deleted rnfCDGEAB gene cluster; ΔrseC, C. ljungdahlii with deleted rseC gene; and Δnar, C. ljungdahlii with deleted nitrate reductase gene cluster. CO_2_, carbon dioxide; and H_2_, hydrogen. Given in percentage is the difference in performance in comparison to the wild type with the same nitrogen source.*

*^a^μ values were calculated based on the individual OD_600_ values of each triplicate in the exponential growth phase.*

*^b^n.d., not detectable.*

*Significant (P ≤ 0.05), ***significant (P ≤ 0.001).

In contrast, the *C. ljungdahlii* ΔRNF strain was unable to grow with carbon dioxide and hydrogen regardless of the nitrogen source (Figure 2A). This was expected and confirmed previous findings in which *rnf* genes were deleted or disrupted (Tremblay et al., 2012; Westphal et al., 2018). We did not observe a pH decrease, and also not an accumulation of ethanol as a metabolic activity of non-growing cultures of *C. ljungdahlii* ΔRNF. However, some minor amounts of acetate were detected, which arguably resulted from the fermentation of the small amount of yeast extract (Figures 2B–D and Table 1). Furthermore, nitrate reduction as a metabolic activity of non-growing cultures was not detectable in *C. ljungdahlii* ΔRNF with carbon dioxide and hydrogen (Figure 2F).

### The Deletion of the *Rhodobacter* Nitrogen Fixation Complex Influenced Nitrate Reduction During Heterotrophy

For the *C. ljungdahlii* ΔRNF strain, heterotrophic growth with fructose was still possible but notably reduced (Figure 1C and Supplementary Figure 1A). Compared to the wild type, we observed significant reduction for *C. ljungdahlii* ΔRNF with ammonium and nitrate, respectively, of the growth rates (−34%, −42%), maximum OD_600_ values (−53%, −56%), and maximum acetate concentrations (−32%, −42%) (Table 1 and Supplementary Table 5). The maximum ethanol concentration was significantly reduced with ammonium (−41%), and ethanol was not produced at all with nitrate (Table 1 and Supplementary Figures 1C,D). During heterotrophy, *C. ljungdahlii* ΔRNF was able to utilize nitrate but considerably slower than the wild type (Supplementary Figure 1F). At the end of the cultivation, cultures of *C. ljungdahlii* ΔRNF had only consumed 49% of the provided nitrate (Supplementary Figure 1F). Overall, we observed a halt in growth and metabolic activity for cultures of *C. ljungdahlii* ΔRNF with fructose after 47 h of cultivation in nitrate-containing medium and after 56 h of cultivation in ammonium-containing medium (Supplementary Figure 1). Fructose concentrations at the end of the cultivation remained at a concentration of 8.0–9.7 mM, which is still 30–35% of the initially provided concentration (Supplementary Figure 1G). The pH did not increase during heterotrophy with nitrate in *C. ljungdahlii* ΔRNF, but instead slowly decreased until the end of the cultivation (Supplementary Figure 1B). Notably, the final pH for heterotrophic cultures of *C. ljungdahlii* ΔRNF with nitrate was still higher compared to cultures with ammonium (Supplementary Figure 1B).

### The *rseC* Gene Is Essential for Autotrophy in *Clostridium ljungdahlii*

Next, we investigated the role of the small putative regulator gene *rseC* (CLJU_c11350). We applied our CRISPR-Cas12a system to delete the *rseC* gene from the genome (Supplementary Figure 2A). We performed growth experiments with the generated *C. ljungdahlii* Δ*rseC* strain under the same conditions as for the *C. ljungdahlii* WT and ΔRNF strains. Cultures of *C. ljungdahlii* Δ*rseC* did not grow with carbon dioxide and hydrogen, neither with ammonium nor with nitrate, during a total cultivation time of 189 h (Figure 2). Non-growing cultures for this strain did not accumulate notable concentrations of acetate or ethanol during the cultivation time (Figures 2C,D). Furthermore, we did not observe nitrate reduction or a remarkable change in pH as a metabolic activity of non-growing cultures for this strain during autotrophy (Figures 2B,E,F).

However, heterotrophic growth of *C. ljungdahlii* Δ*rseC* was possible, and in contrast to *C. ljungdahlii* ΔRNF, the impact was less pronounced for growth with ammonium but limited to some extent with nitrate (Supplementary Figure 1A). In comparison to the wild type with ammonium, only the maximum ethanol concentration (−29%) was significantly reduced (Supplementary Table 5 and Supplementary Figure 1C). In contrast, with nitrate, the heterotrophic growth rates (−34%), maximum OD_600_ (−30%), and maximum ethanol concentrations (−42%) were all significantly reduced, while the maximum acetate concentration was not (Supplementary Table 5 and Supplementary Figure 1D). Nitrate reduction was not restricted during heterotrophy in *C. ljungdahlii* Δ*rseC* (Supplementary Figures 1E,F). Indeed, we observed a rapid utilization of all supplied nitrate within 60 h of cultivation, which is similar to the observations that we had made for the wild type (Supplementary Figure 1F). Thus, *rseC* seems to be involved in regulating the expression of the RNF-gene cluster during autotrophy, but not during heterotrophy. However, the exact impact on gene expression of the RNF-gene cluster cannot be deduced from these findings.

### Plasmid-Based Complementation Relieved the Phenotypes of the *Clostridium ljungdahlii* ΔRNF and Δ*rseC* Strains

We questioned whether the wild-type phenotype, particularly with respect to autotrophy, can be restored by plasmid-based gene complementation in the *C. ljungdahlii* ΔRNF and *C. ljungdahlii* Δ*rseC* strains. Therefore, we generated the plasmid-carrying strains *C. ljungdahlii* ΔRNF pMTL83151_P_nat__*rnfCDGEAB* and *C. ljungdahlii* Δ*rseC* pMTL83152_*rseC*. The plasmids encode the RNF-gene cluster under the control of the native promoter region upstream of the *rnfC* gene from the genome (P_nat_) in pMTL83151_P_nat__*rnfCDGEAB* and the *rseC* gene under the control of the constitutive thiolase promoter (P*_*thl*_*) in pMTL83152_*rseC*, respectively. We investigated the complementation strains in ammonium-containing medium with carbon dioxide and hydrogen for growth (Supplementary Figure 3). Indeed, the plasmid-based expression of the deleted genes relieved the phenotype and enabled autotrophy with carbon dioxide and hydrogen for both strains (Table 2 and Supplementary Figure 3). The control strains that carried an empty plasmid failed to grow autotrophically, as we had already observed for the non-complemented deletion strains.

**TABLE 2:** Performance of the plasmid-based complemented deletion strains of *C. ljungdahlii* in autotrophic batch cultivation experiments.

Strain	Nitrogen source	Growth rate (μ in h^–1^)^a^	Maximum OD_600_ value	Maximum acetate concentration (mM)	Maximum ethanol concentration (mM)
WT	Ammonium	0.024 ± 0.002	0.56 ± 0.01	59.5 ± 1.8	1.9 ± 0.4
ΔRNF compl.	Ammonium	0.024 ± 0.001 (−3%, **)	0.40 ± 0.03 (−29%,**)	46.7 ± 3.7 (−22%, **)	2.2 ± 0.2 (+18%, n.s.^c^)
Δ*rseC* compl.	Ammonium	0.022 ± 0.002 (−8%, n.s.**^c^**)	0.66 ± 0.03 (+17%, *)	63.2 ± 0.2 (+6%, *)	n.d.^b^
WT	Nitrate	0.072 ± 0.004	1.00 ± 0.06	50.1 ± 2.1	8.0 ± 1.6
Δ*nar* compl.	Nitrate	0.054 ± 0.001 (−26%, **)	1.54 ± 0.03 (+ 54%, ***)	41.7 ± 2.5 (−17%, *)	3.4 ± 0.5 (−58%, *)

*Cultures were grown with carbon dioxide and hydrogen (autotrophy) in PETC medium, which contained either ammonium or nitrate as nitrogen source. A gas atmosphere of H_2_/CO_2_ (80/20 vol-%) with 0.5 bar overpressure was applied. Data is represented as mean values from biological triplicates ± standard deviation. The WT data from Table 1 are shown again for comparison. WT, wild type; ΔRNF, deletion of the rnfCDGEAB gene cluster; ΔrseC, deletion of the rseC gene; Δnar, deletion of the nitrate reductase gene cluster; ΔRNF compl., complementation strain C. ljungdahlii pMTL83151_P_nat__rnfCDGEAB; ΔrseC compl., complementation strain C. ljungdahlii pMTL83152_rseC; and Δnar compl., complementation strain C. ljungdahlii pMTL83152_nar. Given in percentage is the difference in performance in comparison to the wild type with the same nitrogen source.*

*^a^μ values were calculated based on the individual OD_600_ values of each triplicate in the exponential growth phase.*

*^b^n.d., not detectable.*

*^c^n.s., not significant (P > 0.05).*

*Significant (P ≤ 0.05), **significant (P ≤ 0.01), ***significant (P ≤ 0.001).

**FIGURE 3:**
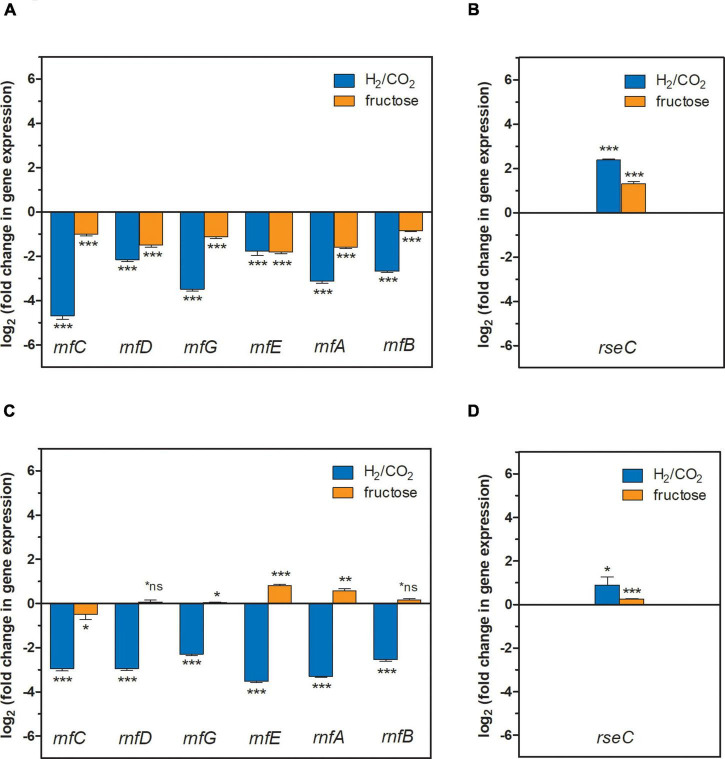
Gene expression change of the *rnfCDGEAB* cluster genes and the *rseC* gene in the ΔRNF and Δ*rseC* deletion strains. **(A)** Gene expression change for the genes *rnfC*, *rnfD*, *rnfG*, *rnfE*, *rnfA*, and *rnfB* in strain *C. ljungdahlii* Δ*rseC* after 3 h cultivation time; **(B)** gene expression change for the gene *rseC* in strain *C. ljungdahlii* ΔRNF after 3 h cultivation time; **(C)** gene expression change for the genes *rnfC*, *rnfD*, *rnfG*, *rnfE*, *rnfA*, and *rnfB* in strain *C. ljungdahlii* Δ*rseC* after 20 h cultivation time; and **(D)** gene expression change for the gene *rseC* in strain *C. ljungdahlii* ΔRNF after 20 h cultivation time. RNA samples were purified from cultures that were cultivated either autotrophically with hydrogen and carbon dioxide (blue bars) or heterotrophically with fructose (orange bars). cDNA was synthesized from the purified RNA samples and used as template for qRT-PCR analyses. The individual gene expression profiles of each gene was calculated using the wild-type strain as reference, which was grown under the same conditions. The *rho* gene was used as “housekeeping” gene. The fold change in gene expression was determined with the 2^–ΔΔCT^ method (Livak and Schmittgen, 2001). ***P ≤ 0.001; **P ≤ 0.01; *P ≤ 0.05; *ns, not significant (*P* > 0.05). We defined log_2_ (fc) ≤ –2 as downregulated genes and ≥ + 2 as upregulated genes.

For *C. ljungdahlii* ΔRNF pMTL83151_P_nat__*rnfCDGEAB* we found a significantly reduced maximum OD_600_ (−71%), and maximum acetate concentration (−22%) (Table 1 and Supplementary Figures 3A–C), while the maximum ethanol concentration was similar to the wild type (Supplementary Figure 3D). Furthermore, the complemented strain had a prolonged *lag* phase of 71 h (Supplementary Figure 3A). Notably, the medium for the complementation experiments always contained antibiotics, which generally caused a slightly negative impact on growth of plasmid-carrying *C. ljungdahlii* strains such as just described for the *C. ljungdahlii* ΔRNF pMTL83151_P_nat__*rnfCDGEAB* strain. Surprisingly, this was not the case for the *C. ljungdahlii* Δ*rseC* pMTL83152_*rseC* strain. Instead of a prolonged *lag* phase, we observed a slightly shortened *lag* phase for this strain (Figures 1A, 3A). Compared to the wild type, this strain reached a slightly but significantly increased maximum OD_600_ (+17%) and maximum acetate concentration (+6%) (Table 2 and Supplementary Figure 3A). However, this strain did not produce any detectable ethanol during the cultivation (Table 2 and Supplementary Figure 3D). Furthermore, the pH value did not show any notable change (Supplementary Figure 3B). The differences in the performance of our complementation strains might be explained by the plasmid copy number and the differences in the used promoter sequences for the complementation plasmids, while this hypothesis awaits further experimentation (Supplementary Text 1D).

### The Gene Expression Profiles of *rnf* Genes and the *rseC* Gene in the Deletion Strains Revealed Regulatory Effects

We further investigated the activating or repressing function on the gene expression of the RNF-gene cluster by RseC. For this, we performed qRT-PCR analyses to investigate the individual expression profiles of the genes *rnfC, rnfD, rnfG, rnfE, rnfA, rnfB*, and *rseC* in the *C. ljungdahlii* Δ*rseC* strain. We included the *C. ljungdahlii* ΔRNF and *C. ljungdahlii* WT strains as controls (Supplementary Figure 5, section “Materials and Methods”). We analyzed samples after 3 and 20 h to investigate the transcriptomic response after inoculating the autotrophic and heterotrophic main cultures from heterotrophic pre-cultures. During the cultivation of the six main cultures (three strains, two conditions), *C. ljungdahlii* WT grew during autotrophy and heterotrophy, while *C. ljungdahlii* ΔRNF and *C. ljungdahlii* Δ*rseC* only grew during heterotrophy (Supplementary Figures 5C,D). Thus, we exposed non-growing cells of *C. ljungdahlii* ΔRNF and *C. ljungdahlii* Δ*rseC* to an autotrophic environment and collected samples after 3 and 20 h. We argued that this would result in a transcriptomic response, even though the cultures did not show a difference in OD_600_ over the 20-h incubation time of this experiment (*C. ljungdahlii* ΔRNF, 0.20 ± 0.02 and *C. ljungdahlii* Δ*rseC*, 0.22 ± 0.02, respectively), but remained at the inoculation OD_600_. In comparison, for the wild type, 1–2 generations can be expected within 20 h of cultivation during autotrophy, which resulted in an increase of the OD_600_ from 0.28 to 0.39 (Supplementary Figure 5C).

**FIGURE 4:**
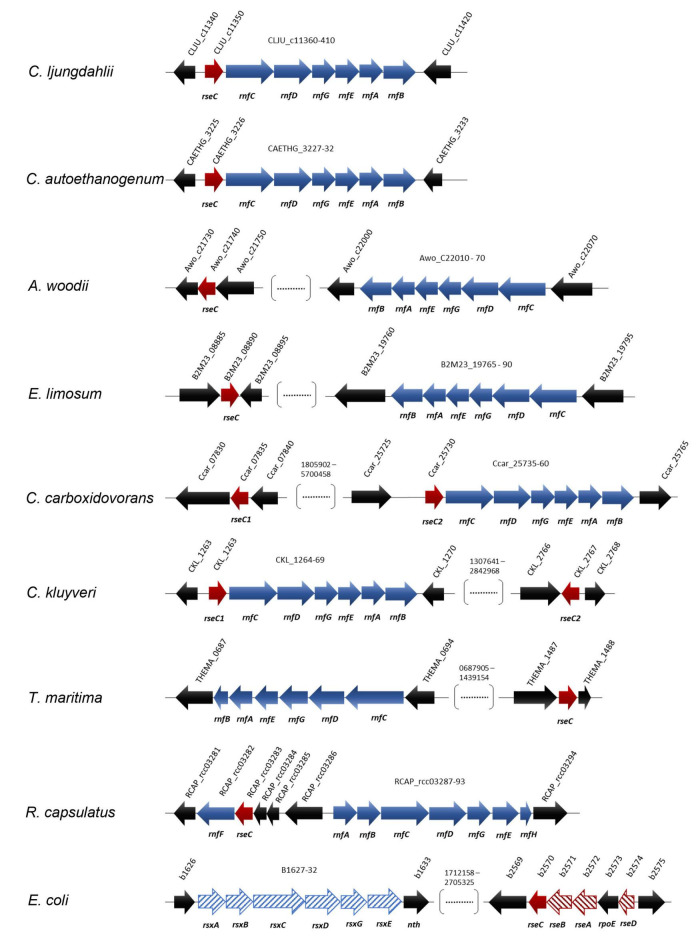
Location and orientation of *rseC* genes in model microbes that possess RNF complex gene clusters. The conserved protein domain RseC_MucC (pfam04246) was identified in the *rseC* protein sequence of *C. ljungdahlii* and used to search for putative *rseC* genes in the genome of *C. autoethanogenum, A. woodii*, *E. limosum, C. carboxidovorans, C. kluyveri, T. maritima, R. capsulatus*, and *E. coli*. All sequence analyses and gene arrangements were adapted from the JGI platform and the NCBI database (03/2021). The type strains are listed in Table 3. In red, putative *rseC* genes; in red pattern fill, *rseC*-associated genes in *E. coli*; in blue, RNF-complex gene cluster; in blue pattern fill, *rsx* genes, which are homologous to the *rnf* genes in *R. capsulatus.*

The qRT-PCR results in this paragraph are given as log_2_ (fold change in gene expression), where we discuss a value of ≤ −2 (0.25-fold) as a biologically relevant downregulation, and a value of ≥ + 2 (fourfold) as a biologically relevant upregulation (Figure 3). We did not measure any expression signals for any of the deleted RNF genes in the *C. ljungdahlii* ΔRNF strain and for the deleted *rseC* gene in the *C. ljungdahlii* Δ*rseC* strain. We found that all RNF-gene cluster genes (except for *rnfE*, −1.8 ± 0.2) were downregulated [ranging from −2.2 ± 0.1 (*rnfD*) to −4.7 ± 0.1 (*rnfC*)] in the *C. ljungdahlii* Δ*rseC* strain, when exposing non-growing cells of this strain to hydrogen and carbon dioxide (Figure 3). We observed a similar pattern of downregulation for the 3 and 20-h samples of the *C. ljungdahlii* Δ*rseC* strain (Figures 3A, 4C). In the heterotrophic samples, the RNF-gene cluster genes were not downregulated in the 3-h samples [ranging from −1.0 ± 0.1 (*rnfC*) to −1.8 ± 0.1 (*rnfE*)]. After 20 h of cultivation time during heterotrophy, we even observed a pattern in which none of the genes had a log2 (fold change in gene expression) of ≤ −1 or ≥ + 1. In the *C. ljungdahlii* ΔRNF strain as a control, we found that *rseC* expression was upregulated in the 3-h samples during autotrophy (+2.4 ± 0.04) but not during heterotrophy (+1.3 ± 0.1) (Figure 3B). In the 20-h samples both conditions were not different (Figure 3D). For the wild type, all genes (except for *rnfD* and *rnfE* in the 3-h sample) were upregulated during autotrophy when compared to heterotrophy for the 3-h samples [ranging from + 2.3 ± 0.03 (*rnfB*) to + 5.4 ± 0.1 (*rseC*)], and for the 20-h samples [ranging from + 2.8 ± 0.04 (*rnfB*) to + 3.8 ± 0.1 (*rnfE*)], respectively (Supplementary Figure 5). Thus, from our qPCR results we concluded that RseC likely has a positive regulatory impact on the RNF-gene cluster during autotrophy, but not during heterotrophy, while we cannot rule out that this regulation was an indirect effect, which involves further regulatory elements, from these experiments.

### The *rseC* Gene Is Abundantly Found Among Acetogens

Based on our previous findings, we investigated whether *rseC* genes are present in the genomes of selected other microbes, including the most prominent model acetogens. We searched for putative *rseC* genes in the RNF-complex gene-containing genomes of the model acetogens *C. ljungdahlii*, *Clostridium autoethanogenum*, *A. woodii*, *Eubacterium limosum*, *Clostridium carboxidovorans*, and the model non-acetogen *Clostridium kluyveri* (Figure 4 and Table 3). Indeed, we found putative *rseC* genes for all these candidates. We looked at the genomic location and distance of the *rseC* gene to the RNF-gene cluster (Figure 4 and Table 3). We noticed that the *rseC* gene was located directly upstream of the RNF complex gene cluster in *C. ljungdahlii* (CLJU_c11350), *C. autoethanogenum* (CAETHG_3225), *C. carboxidovorans* (Ccar_25725), and *C. kluyveri* (CKL_1263). However, the *rseC* gene in *A. woodii* (Awo_C21740) and *E. limosum* (B2M23_08890) was not in direct genetic vicinity of the RNF-gene cluster (Figure 4 and Table 3). In addition, we identified a second gene with homologies to *rseC* in *C. carboxidovorans* (Cca_07835) and *C. kluyveri* (CKL_2767), but neither RNF-complex genes nor other genes that are involved in the autotrophic metabolism, such as the genes for the Wood-Ljungdahl pathway, are located in the direct vicinity of these second *rseC* homologs (Figure 4 and Table 3). Furthermore, the RseC protein sequence seems to be highly conserved in the microbes that contain an RNF-gene cluster (Supplementary Figure 6 and Supplementary Text 1F).

**TABLE 3:** Distribution of *rseC* genes in model microbes.

Microbe^a^	Amount of *rseC* genes^b^	RNF or Ech	*rseC* associated with RNF genes	Gene locus
*Clostridium ljungdahlii*	1	RNF^c^	Yes	CJLU_c11350
*Clostridium autoethanogenum*	1	RNF^c^	Yes	CAETHG_3226
*Clostridium carboxidovorans*	2	RNF^c^	Yes, one of them	Ccar_07835, Ccar_025730
*Clostridium kluyveri*	2	RNF^c^	Yes, one of them	CKL_1263, CKL_2767
*Eubacterium limosum*	1	RNF^d^	No	B2M23_08890
*Acetobacterium woodii*	1	RNF^d^	No	Awo_c21740
*Thermotoga maritima*	1	RNF^d^	No	THEMA_1487
*Moorella thermoacetica*	0	Ech	No	−
*Thermoanaerobacter kivui*	0	Ech	No	−
*Rhodobacter capsulatus*	1	RNF^e^	Yes	RCAP_rcc03283
*Escherichia coli*	1	Rsx^f^	No, but with Rsx	b2570

*^a^The type strains were: C. ljungdahlii DSM13528; C. autoethanogenum DSM10061; C. carboxidovorans P7; C. kluyveri DSM555; E. limosum ATCC8486; A. woodii DSM1030; T. maritima DSM3109; M. thermoacetica ATCC39073; T. kivui DSM2030; R. capsulatus SB1003; and E. coli K-12.*

*^b^The pfam domain pfam04426 was used to search for putative rseC genes in each genome.*

*^c^The RNF complex uses (or is supposed to use) protons.*

*^d^The RNF complex uses (or is supposed to use) sodium ions.*

*^e^The RNF complex either uses protons or sodium ions. Experimental data are missing.*

*^f^Rsx is encoded by rsxABCDGE and is homologous to the RNF-gene cluster in R. capsulatus.*

In contrast, we did not find a putative *rseC* gene when we searched the genomes of two further model acetogens *Moorella thermoacetica* and *Thermoanaerobacter kivui*, which possess an energy-converting hydrogenase (Ech) complex instead of an RNF complex (Hess et al., 2014). Notably, we also identified a putative *rseC* gene in the non-acetogenic bacteria *R. capsulatus*, which is the microbe in which the RNF complex was first described (Schmehl et al., 1993), and in *Thermotoga maritima* (Schmehl et al., 1993). The *rseC* gene in *R. capsulatus* is located upstream of *rnfF* instead of *rnfC*, which is separated by five genes (Figure 4). In *T. maritima*, RNF genes were not found next to the putative *rseC* gene (THEMA_1487) (Figure 4). Also *E. coli* possesses one *rseC* gene that is organized in the *rseABC* gene cluster, which plays an important role in the SoxR-mediated oxidative stress response as described elsewhere (Figure 4 and Supplementary Text 1E; Koo et al., 2003).

These findings led to the question, whether we can find an acetogen that contains an RNF-complex but no *rseC* gene. To answer this question, we performed a more extensive and automated genome search. We based this search on a collection of 47 out of 61 acetogens with a fully sequenced genome from Bengelsdorf et al. (2018), which are stored in the German Collection of Microorganisms and Cell Cultures (*Deutsche Sammlung von Mikroorganismen und Zellkulturen, DSMZ)* and American Type Culture Collection (ATCC). In this automated genome search, we confirmed our initial findings, and in total identified 30 acetogens that contained potential RNF-complex genes (Supplementary Table 7). Seven of these acetogens (*Acetitomaculum ruminis DSM 5522, Blautia hydrogenotrophica DSM 10507, Blautia schinkii, Marvinbryantia formatexigens DSM 14469, Oxobacter pfennigii, Terrisporobacter mayombei*, and *Treponema primitia ZAS-2*) did not show evidence for an *rseC* gene. We identified several acetogens that contained more than one potential *rseC* gene (Supplementary Table 7). Interestingly, we found three acetogens (*Acetobacterium fimetarium*, *Clostridium magnum* DSM 2767, and *Oxobacter pfennigii*), which have both potential RNF-complex genes and Ech-complex genes in their genomes, while of those *Oxobacter pfennigii* was not found to contain an *rseC* gene as mentioned above (Supplementary Table 7).

Overall, we found with our genome search that not all investigated acetogens that contain RNF genes have putative *rseC* genes in their genomes. Some acetogens have several putative *rseC* homologs, and some of these putative *rseC* genes are associated with the RNF genes, while others are not. We did not identify an acetogen that contained an *rseC* gene and exclusively an Ech-complex but no RNF-complex (Table 3 and Supplementary Table 7). The reasons for these observations and the regulatory effects in acetogens with and without an *rseC* gene will be important future research questions.

### The *nar* Gene Cluster Encodes a Functional Nitrate Reductase in *Clostridium ljungdahlii*

**FIGURE 5:**
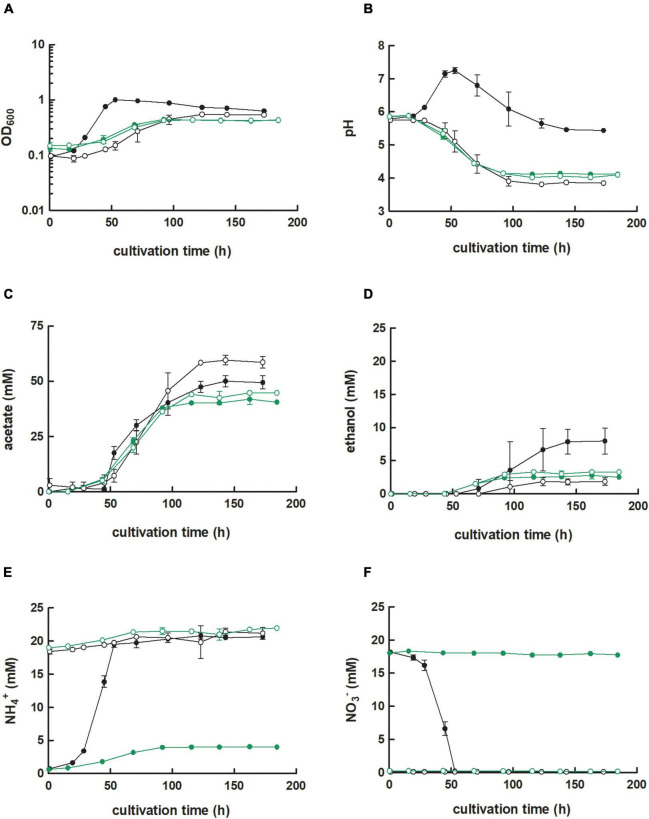
Growth, pH behavior, nitrate reduction of *C. ljungdahlii* Δ*nar* with H_2_ and CO_2_. Cultures were grown in 100 mL PETC medium in 1 L bottles at 37°C and 150 rpm for 185 h. The headspace consisted of H_2_ and CO_2_ (80/20 vol-%) and was set to 0.5 bar overpressure. The medium contained either 18.7 mM nitrate (NO_3_^–^) (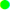) or 18.7 mM ammonium (NH_4_^+^) (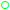) as nitrogen source. The *C. ljungdahlii* WT data (●, ◦) from Figure 1 is given for comparison. All cultures were grown in biological triplicates, data is given as mean values, with error bars indicating the standard deviation. **(A)** Growth; **(B)** pH-behavior; **(C)** acetate concentrations; **(D)** ethanol concentration; **(E)** ammonium concentration; and **(F)** nitrate concentrations. Δ*nar*, deletion of nitrate reductase gene cluster; rpm, revolutions per minute; CO_2_, carbon dioxide; and H_2_, hydrogen.

**FIGURE 6:**
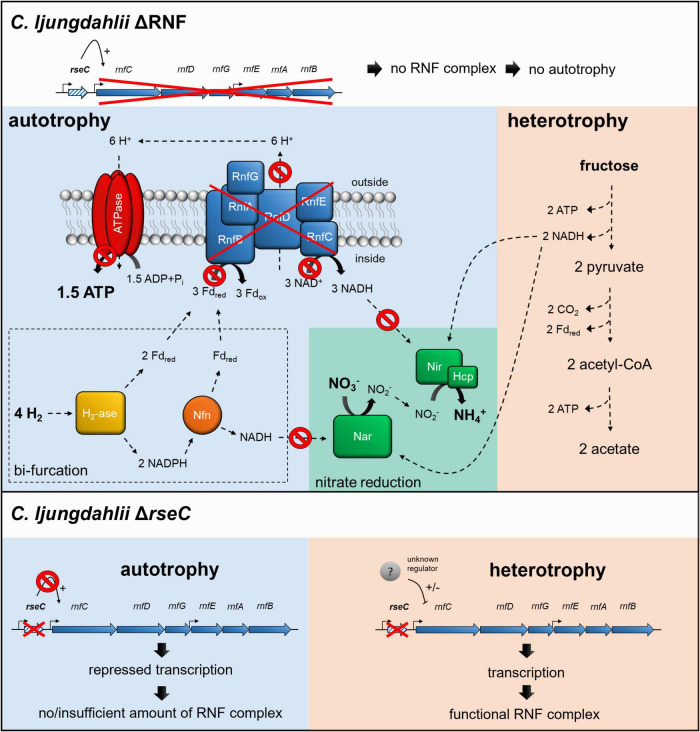
Schematic model of RNF-gene regulation and nitrate reduction in the deletion strains *C. ljungdahlii* ΔRNF and *C. ljungdahlii* Δ*rseC* during autotrophy and heterotrophy. In both deletion strains, nitrate reduction is not possible in non-growing cells during autotrophy with carbon dioxide and hydrogen due to the lack of a functional RNF complex, and thus the missing regeneration of reducing equivalents such as NADH. On the contrary, nitrate reduction can proceed in *C. ljungdahlii* ΔRNF during heterotrophy with NADH, which is provided by glycolysis of fructose. In *C. ljungdahlii* Δ*rseC*, the RNF complex genes are repressed during autotrophy but not during heterotrophy, which indicates a further unknown regulation mechanism during heterotrophy. Thus, a functional RNF complex is formed, and nitrate reduction can proceed such as proposed for the wild type. H_2_, hydrogen; H^+^, proton, CO_2_, carbon dioxide; NO_3_^–^, nitrate; NO_2_^–^, nitrite; NH_4_^+^, ammonium; ATP, adenosine triphosphate; ADP + P_i_, adenosine diphosphate + phosphate; Fd_red/ox_, reduced/oxidized ferredoxin; NADH/NAD^+^, reduced/oxidize nicotinamide adenine dinucleotide; NADPH/NADP^+^, reduced/oxidized nicotinamide adenine dinucleotide phosphate; RnfCDGEAB, RNF-complex subunits; Nar, nitrate reductase; Nir, nitrite reductase; Hcp, hydroxylamine reductase; H_2_-ase, bifurcating hydrogenase/lyase; Nfn, bifurcating transhydrogenase; e^–^, electron; ΔRNF, *C. ljungdahlii* ΔRNF; and Δ*rseC*, *C. ljungdahlii* Δ*rseC*. The model was adapted from Emerson et al. (2019).

We had found that nitrate reduction during heterotrophy is impacted for the *C. ljungdahlii* ΔRNF strain but not the *C. ljungdahlii* Δ*rseC* strain. Thus, we aimed to explore nitrate metabolism and the interplay with the RNF complex further. For *C. ljungdahlii*, it was postulated that nitrate is reduced by nitrate reductase to nitrite and, subsequently, converted *via* nitrite reductase and hydroxylamine reductase into ammonium, and the involved genes were predicted in the genome (Köpke et al., 2010; Nagarajan et al., 2013). Emerson et al. (2019) had found that in the presence of nitrate the expression level of the genes that encode the putative nitrate reductase (CLJU_c23710-30) were significantly increased. The three genes are annotated as nitrate reductase NADH oxidase subunit (CLJU_c23710), nitrate reductase electron transfer subunit (CLJU_c23720), and nitrate reductase catalytic subunit (CLJU_c23730) (Köpke et al., 2010). We refer to these three genes (CLJU_c23710-30) as the *nar* gene cluster. We verified the absence of the *nar* gene cluster from the genome of the *C. ljungdahlii* Δ*nar* strain, after mediating the deletion with our CRISPR-Cas12a system (Supplementary Figure 2B). This strain was able to grow during autotrophy and heterotrophy, but had completely lost the ability to reduce nitrate under both conditions (Figure 5F and Supplementary Figure 7F). Consequently, we observed similar growth and pH behavior for cultures of *C. ljungdahlii* Δ*nar* during autotrophy with either ammonium or nitrate (Figures 5A, 6B and Supplementary Figures 7A,B). Enhanced autotrophic growth in nitrate-containing medium when compared to ammonium-containing medium, such as with the wild-type strain, was not detected (Figure 5A).

However, we still observed differences in the growth when compared to the wild type. For ammonium and nitrate conditions, respectively, growth rates (−24%, −76%), maximum OD_600_ (−21%, −55%), and maximum acetate concentrations (−25%, −16%) during autotrophy of *C. ljungdahlii* Δ*nar* were significantly reduced (Table 1 and Figure 5A). Instead, the maximum ethanol concentrations were significantly increased (+79%) for ammonium, but significantly decreased (−64%) for nitrate conditions, respectively (Figure 5D and Table 1). A pH increase as a consequence of ammonium production from nitrate reduction, such as observed for the wild type, was not observed in cultures of *C. ljungdahlii* Δ*nar* (Figure 5B).

For heterotrophic cultures with ammonium, we did not observe significant differences in growth behavior and acetate production to the wild type. Only the maximum ethanol concentration was significantly increased (+45%) (Supplementary Table 5, Supplementary Figure 7). With fructose and nitrate, the maximum observed OD_600_ (−32%) and maximum acetate concentration (−34%) were significantly reduced, while the maximum ethanol concentration (+234%) was significantly increased (Supplementary Table 5 and Supplementary Figure 7D). The provided fructose was only consumed completely by *C. ljungdahlii* Δ*nar* in ammonium-containing but not in nitrate-containing medium (Supplementary Figure 7G).

Finally, we confirmed that the complementation of *C. ljungdahlii* Δ*nar* with the plasmid pMTL83152_*nar*, which encodes the *nar* gene cluster under the expression control of the constitutive P*_*thl*_* promoter, enabled the *C. ljungdahlii* Δ*nar* pMTL83152_*nar* strain to utilize nitrate under autotrophic conditions again, while this was not possible in an empty plasmid control strain (Supplementary Figure 8). The nitrate cultures of *C. ljungdahlii* Δ*nar* pMTL83152_*nar* had significantly different growth rates (−26%), maximum OD_600_ (+54%), as well as maximum acetate (−17%) and ethanol (−57%) concentrations in comparison to the wild type when growing with nitrate (Table 2 and Supplementary Figure 8). In summary, we revealed that the expression of the *nar* gene cluster led to the only functional nitrate reductase in *C. ljungdahlii* under the tested conditions.

## Discussion

### A Functional *Rhodobacter* Nitrogen Fixation Complex Is Essential for Autotrophy but Not for Heterotrophy in *Clostridium ljungdahlii*

Here, we provided further insight into the autotrophy of *C. ljungdahlii* and the connection to nitrate metabolism. With the strain *C. ljungdahlii* ΔRNF, we confirmed previous work by Tremblay et al. (2012) that the absence of the RNF complex leads to a complete loss of autotrophy in *C. ljungdahlii*. Unlike in the previous study by Tremblay et al. (2012), our strain provides a stable genotype that cannot revert back to the wild-type genotype, which can be used to further study the energy conservation principles in this acetogen (Figures 1B, 2). Heterotrophic growth in our deletion strain was still possible, but considerably reduced when compared to the wild type (Figure 1C and Supplementary Figure 1), which was expected from the work by Tremblay et al. (2012). While we did not measure the difference in the headspace gas composition during heterotrophy for *C. ljungdahlii* ΔRNF and wild type, we argue that *C. ljungdahlii* ΔRNF lost the ability to fixate the carbon dioxide that is released during glycolysis, which is the defining feature of acetogens (Drake et al., 2008; Schuchmann and Müller, 2014). Thus, even though the Wood-Ljungdahl pathway was still present, this strain was not able to balance the electrons in the metabolism sufficiently to drive the Wood-Ljungdahl pathway and/or in other metabolic pathways, which resulted in a remaining 30% of fructose that was not consumed until the end of the batch cultivation (Supplementary Figure 1). However, further research is required to confirm this hypothesis. The RNF deletion in *A. woodii* did also lead to reduced acetate production during heterotrophy, but the strain reached similar OD_600_ values compared to the *A. woodii* wild type (Westphal et al., 2018). In comparison to *C. ljungdahlii*, the RNF complex of *A. woodii* uses sodium ions instead of protons to generate the chemiosmotic gradient, which is then consumed by a sodium-dependent F_1_F_O_ ATPase to generate ATP (Biegel and Müller, 2010; Hess et al., 2013). Overall, this further confirms the meticulous differences in the energy conservation and redox balancing in different acetogens (Katsyv and Müller, 2020), which have to be considered to apply acetogens for biotechnological purposes.

### RseC Is a Regulator of the *Rhodobacter* Nitrogen Fixation Complex Genes and Plays a Critical Role During Autotrophy

We further investigated the regulation of the RNF-gene cluster by the putative regulator RseC. The *rseC* gene is known to encode a transcriptional regulator in other microbes such as *E. coli* and *S. Typhimurium* (Supplementary Text 1E; Beck et al., 1997; De Las Peñas et al., 1997; Yura and Nakahigashi, 1999; Koo et al., 2003). Our results demonstrated that RseC played a critical role for the formation of a functional RNF complex in *C. ljungdahlii* (Table 2 and Figure 2). A deletion of the *rseC* gene led to the complete loss of autotrophy (Figure 2). With our qRT-PCR analyses, we confirmed that RseC, indeed, had a positive regulatory effect on the expression of the RNF-gene cluster during autotrophy. Our results indicate that RseC is essential for the activation of RNF-gene cluster expression during autotrophy, but not during heterotrophy, while we cannot rule out that this activation is mediated by other modulating activities such as secondary regulators (Figures 3A,C, 6, Supplementary Figure 5, and Supplementary Text 1G). Al-Bassam et al. (2018) identified a transcription start site upstream of *rseC* and found that *rseC* was poorly translated under heterotrophic conditions similar to *rnfC* in *C. ljungdahlii*. Two transcription start sites were identified within the RNF-complex gene cluster *rnfCDGEAB*, which could be preceded by potential promoter-binding sites for RseC (Al-Bassam et al., 2018). We can speculate that RseC most likely binds to the transcription start site, which is located upstream of the *rnfC* gene, because the entire RNF-gene cluster was downregulated during autotrophy and heterotrophy after 3 h of cultivation (Figure 3A). Based on our qRT-PCR results from the heterotrophic samples after 20 h of cultivation, it can be further speculated that the second transcription start site, which is located upstream of *rnfE* (Al-Bassam et al., 2018), might be a second binding site for RseC. We see a slight upregulation of the three genes *rnfE, rnfA*, and *rnfB* in these samples for the *rseC* deletion compared to the wild-type strain. Thus, we argue that RseC could act as a negative regulator to modulate RNF-gene cluster expression during heterotrophy by repressing the genes *rnfE, rnfA*, and *rnfB* (Figure 3C). The plasmid-based complementation of *rseC* in the *C. ljungdahlii* Δ*rseC* strain (and overexpression in the wild-type background) re-enabled growth with carbon dioxide and hydrogen, and reduced the *lag* phase during the transition from heterotrophy to autotrophy (Table 2 and Supplementary Figures 3, 4). This further argues for a function of RseC as a positive regulator of the RNF-gene cluster. The RseC protein in *E. coli* seems to contain two transmembrane domains with the C-terminal end being located in the cytoplasm (Daley et al., 2005). Thus, another possible function of RseC could be the modulation of protein-protein interactions with the RNF complex, because for all the RseC homologs that we investigated here, two transmembrane helices were predicted (Supplementary Table 6). For instance, RseC could stabilize the RNF complex in the membrane, which is required for the electron translocation mechanism or the interaction with other cytoplasmic proteins during autotrophy, but not during heterotrophy. This could explain why a lack of the RseC protein is not leading to the same reduced heterotrophy as observed for the *C. ljungdahlii*ΔRNF strain. The upregulation of the *rseC* gene in the *C. ljungdahlii*ΔRNF strain indicates that the *rseC* gene itself is also under further transcriptional control (Figures 3B,D). An upregulation of the *rseC* gene in all samples might indicate the effort of the cells to induce RNF-gene cluster expression further, which is apparently not possible in the *C. ljungdahlii*ΔRNF strain. Thus, potentially additional direct or indirect regulatory effects are mediated by RseC. This could be, for example, the regulation of further genes as a positive or negative regulator, which in turn could have an effect on the functionality of the RNF complex. Importantly, others had identified a TetR-family protein that is involved as an alternative sigma-factor in the regulation of autotrophy vs. heterotrophy in *C. autoethanogenum* (Lemgruber et al., 2019). Lemgruber et al. (2019) had identified a promoter sequence upstream of the *rseC* gene in *C. autoethanogenum* (CAETHG_3226) that is likely recognized by this alternative sigma-factor. The same sequence motif is found upstream of the *rseC* gene (CJLU_c11350) in *C. ljungdahlii*. However, the function of the TetR-family protein has not yet been investigated in *C. ljungdahlii*. Therefore, protein-DNA binding experiments should be performed to investigate the binding ability of RseC to putative promoter regions, as well as the regulation of the *rseC* gene itself, in future experiments. In addition, the study of the subcellular localization of RseC will be required to unravel the regulatory functions of RseC in *C. ljungdahlii* and other acetogens with an RNF complex in more detail.

### Nitrate Reduction Does Not Require a Functional *Rhodobacter* Nitrogen Fixation Complex but Benefits From a Correct Electron Balance

Furthermore, we investigated the nitrate metabolism in *C. ljungdahlii*. We confirmed that the genes CLJU_c23710-30 encode the functional subunits of the only nitrate reductase under the tested conditions for *C. ljungdahlii* (Figure 5 and Supplementary Figures 7, 8). In the presence of nitrate, the wild type quickly utilized all nitrate even though we had found in all our growth experiments that a sufficient amount of nitrogen-source was covered by the added yeast extract (Figure 2F and Supplementary Figure 1). Thus, nitrate reduction in *C. ljungdahlii* is mainly used for energy conversion, and therefore must be of a dissimilatory function (Emerson et al., 2019). However, *C. ljungdahlii* neither possesses genes for cytochromes nor for the biosynthesis of ubiquinone, which limits the generation of a chemiosmotic gradient to the RNF complex (Köpke et al., 2010). The nitrate reductase in *C. ljungdahlii* is most likely located in the cytosol rather than associated with the membrane, as one would expect from dissimilatory nitrate reductases in bacteria (Zumft, 1997; Köpke et al., 2010; Nagarajan et al., 2013). This type of nitrate reduction was described as fermentative nitrate reduction and was already observed for other microbes, but is less understood than the assimilatory and dissimilatory nitrate reduction in the microbial world (Hall, 1973; Hasan and Hall, 1975; Seifritz et al., 1993; Emerson et al., 2019). Contrarily, nitrate reduction does not function as an independent energy-conserving pathway, because autotrophic growth is not possible even with the addition of nitrate (Figure 2). The stoichiometry for nitrate reduction in *C. ljungdahlii* is proposed as follows: 4 H_2_ + 2 H^+^ + NO_3_^–^ + 1.5 ADP + 1.5 P_i_ ⇌ 4 H_2_O + NH_4_^+^ + 1.5 ATP with Δ_*r*_G’_0_ = −150 kJ/mol H_2_ (Thauer et al., 1977; Emerson et al., 2019). This mechanism would require electron bifurcation from the hydrogenases and the activity of the RNF complex, but would then provide ATP completely independent of the Wood-Ljungdahl pathway (or more general, independent of the carbon metabolism) (Buckel and Thauer, 2018; Emerson et al., 2019). Thus, we hypothesized that nitrate reduction in *C. ljungdahlii* requires a functional RNF complex for a correct electron balance. Indeed, non-growing cells of both *C. ljungdahlii* ΔRNF and *C. ljungdahlii* Δ*rseC* were not able to reduce nitrate during autotrophy (Figure 2F). Further biochemical investigations on the enzyme activity under these conditions will have to provide more detailed insight on this hypothesis. However, nitrate reduction still proceeded in both deletion strains during heterotrophy (Supplementary Figure 1F). In *C. ljungdahlii* ΔRNF a functional RNF complex was not present during heterotrophy because the RNF-complex encoding genes were deleted, but the required reducing equivalents for nitrate reduction were likely provided by glycolysis (Figure 6). In contrast, in *C. ljungdahlii* Δ*rseC*, nitrate reduction was not impacted during heterotrophy, because the RNF complex genes were deregulated (expressed) under these conditions and a functional RNF complex was formed (Figures 3, 6). It remains to be answered whether there is a direct interplay between the nitrate reductase and the RNF complex, and whether this interplay is different during heterotrophy and autotrophy.

### The Electron Balance in the Deletion Strains Is Impacted Beyond Nitrate Reduction

In general, the reduced growth indicated that *C. ljungdahlii* ΔRNF was not able to balance the electrons from glycolysis efficiently during heterotrophy. This led to the reduction in biomass and acetate production, while ethanol production was completely absent in heterotrophic cultures of *C. ljungdahlii* ΔRNF. This indicates that reducing power for a further reduction of acetate was not available (Supplementary Table 5 and Supplementary Figure 1D). In the batch experiments of Emerson et al. (2019), *C. ljungdahlii* WT did not produce considerable amounts of ethanol when growing with nitrate (and carbon dioxide and hydrogen). When *C. ljungdahlii* WT was cultivated in pH-controlled bioreactors under continuous conditions, enhanced biomass and increased ethanol production rates were observed (Klask et al., 2020). This observation could not be fully explained yet, but it was assumed that electrons are used concomitantly for the reduction of nitrate and for the reduction of acetate. This distribution of electrons changed in the absence of the nitrate reductase in the *C. ljungdahlii* Δ*nar* strain and higher maximum ethanol concentrations were observed already in batch conditions. Thus, the overall electron balance between fermentation products, besides the loss of nitrate reduction activity, is impacted by the deletion of the *nar* gene cluster. A possible explanation for increased ethanol production of the *C. ljungdahlii* Δ*nar* strain during heterotrophy could be that more reducing equivalents (e.g., NADH, NADPH) are available to the alcohol dehydrogenases (ADHs), which would be used to reduce nitrate in the wild type, and which predominantly catalyze the reduction of acetyl-CoA to ethanol during heterotrophy but not during autotrophy in *C. ljungdahlii* (Schuchmann and Müller, 2014; Richter et al., 2016; Liew et al., 2017). Consequently, less acetate should be produced by the *C. ljungdahlii* Δ*nar* strain. Indeed, the *C. ljungdahlii* Δ*nar* strain produced only 66% (*P* ≤ 0.001) of the acetate concentration that was measured for the wild type, when growing under heterotrophic conditions with nitrate (Supplementary Table 1 and Supplementary Figure 1C). Furthermore, this would argue for NADH or NADPH as the electron donor of the nitrate reductase as proposed by Emerson et al. (2019). On the contrary, we observed increased ethanol concentrations also in the presence of ammonium instead of nitrate as nitrogen source. This might be due to an involvement of the nitrate reductase in other processes, but could also be due to genetic polar effects in the *nar* deletion strain. In addition, nitrate reduction could be regulated and controlled differently during autotrophy and heterotrophy. It remains elusive, how the change in the distribution of electrons affects other NADH-dependent metabolic pathways in more detail. While further research, such as the investigation of intracellular NAD(H)/NADP(H) levels, is required to understand the regulatory mechanisms during autotrophy and the mechanism of energy conservation during nitrate reduction, with this work, we provide a deeper insight into the autotrophic metabolism and nitrate reduction in *C. ljungdahlii*.

## Data Availability Statement

The original contributions presented in the study are included in the article/Supplementary Material, further inquiries can be directed to the corresponding author/s.

## Author Contributions

C-MK and BM designed the experiments and wrote the manuscript. C-MK performed the genetic work, conducted the growth experiments, analyzed the metabolites, performed the *in silico* research for selected model microbes, and analyzed the experimental data. C-MK and BJ performed the qRT-PCR experiments. IC performed the python-based *in silico* analysis of sequenced genomes of acetogenic bacteria. LA and BM supervised the work. All authors edited the manuscript and approved the final version.

## Conflict of Interest

The authors declare that the research was conducted in the absence of any commercial or financial relationships that could be construed as a potential conflict of interest.

## Publisher’s Note

All claims expressed in this article are solely those of the authors and do not necessarily represent those of their affiliated organizations, or those of the publisher, the editors and the reviewers. Any product that may be evaluated in this article, or claim that may be made by its manufacturer, is not guaranteed or endorsed by the publisher.
