# Supplementary material for: Genetic Evidence Reveals the Indispensable Role of the rseC Gene for Autotrophy and the Importance of a Functional Electron Balance for Nitrate Reduction in Clostridium ljungdahlii
Source: Front Microbiol. 2022 May 9;13:887578. doi: 10.3389/fmicb.2022.887578 (PMC9124969; doi:10.3389/fmicb.2022.887578)
Supplement: Supplementary file 1 [file Data_Sheet_1.docx]

Supplementary Material

**Includes:**

Supplementary Text S1A – 1F

Supplementary Figure S1 – S8

Supplementary Tables S1 – S7

**Supplementary Text S1A - Implementing a CRISPR-Cas12a system for *C. ljungdahlii***

For *C. ljungdahlii*, the first CRISPR-based gene-deletion system was implemented with CRISPR-Cas9 (Huang et al., 2016). Later, a CRISPR-Cas12a system was successfully realized in *C. ljungdahlii* (Zhao et al., 2019). Cas12a, also known as Cpf1, is an alternative Cas-type nuclease, which uses an AT-rich protospacer adjacent motif sequence instead of GC-rich protospacer adjacent motif sequences, which are preferred by Cas9. Since genomes of acetogens are in general AT rich (Drake et al., 2008), the utilization of a CRISPR-Cas12a system offers more potential genome-editing sites compared to a CRISPR-Cas9 system. In addition, Cas12a cleaves the targeted DNA in a staggered pattern, which is postulated to increase the efficiency of DNA-repair mechanisms or might allow gene insertion through non-homologous end joining (Fagerlund et al., 2015; Zetsche et al., 2015; Bayat et al., 2018).

We implemented a CRISPR-Cas12a system in the shuttle-vector system pMTL80000 (Heap et al., 2009) (**Figure 1A**). We chose the constitutive thiolase promoter P*_thl_* (Heap et al., 2009) and the anhydrotetracycline-inducible promoter P*_tetR-O1_* (Dong et al., 2012; Woolston et al., 2018), to investigate whether the expression of the Cas12a-nuclease gene itself leads to poor transformation efficiency and genome edit rates as previously reported (Huang et al., 2016). While cloning of the Cas12a gene was readily achieved in *E. coli*, we required several assembling attempts to form the final CRISPR-Cas12a plasmids, which contained the homology-directed repair arms and single guide RNAs. Once the plasmids were generated, we did not observe a noticeable difference between growth of the respective *E. coli* strain or an *E. coli* strain that harbors an empty pMTL plasmid. We also did not see any difference between recombinant *C. ljungdahlii* strains that harbor the control plasmids pMTL83152_Cas12a and pMTL83151_*tetR-O1*_Cas12a, which both lack homologous repair DNA and sgRNAs. However, transfer of the final CRISPR-Cas12a plasmids into *C. ljungdahlii* cells could only be achieved by using conjugation instead of electroporation. Overall, we found successful genome edited cells by using the plasmids pMTL83152_Cas12a-RNF, pMTL83152_Cas12a-rseC, and pMTL83152_Cas12a-nar. All CRISPR-Cas12a plasmids that harbored the P*_tetR-O1_* promoter instead of P*_thl_* were successfully transferred into *C. ljungdahlii* cells, but genome edits were not detectable after induction with anhydrotetracycline and screening several colonies.

**Supplementary Text S1B - Confirmation of strains**

We purified gDNA from the generated strain *C. ljungdahlii* ∆RNF and used it for PCR analyses (***Material and Methods***). A *rnfCDGEAB* fragment could only be amplified when using gDNA from *C. ljungdahlii* WT but not with gDNA of the deletion strain (**Figure 1B**). In addition, the deletion strain showed the expected shortened fragment when using primers that bound outside of the RNF-gene cluster (**Figure 1B**). Sanger sequencing of this fragment confirmed the precise deletion of *rnfCDGEAB* from the genome. We performed similar PCR screening experiments with the *C. ljungdahlii* ∆*rseC* and ∆*nar* strain to confirm the genome edits (**Supplementary Figure S2**). After generating the *C. ljungdahlii* ∆*rseC* strain and verifying the successful genome edit, several transfers in non-selective RCM and a subsequent cultivation at 42°C for 72 h were required to cure the strain from the pMTL83152_Cas12a-rseC plasmid. The genome edit remained stable during this procedure. In contrast, the plasmid curing was achieved quickly for the *C. ljungdahlii* ∆RNF and ∆*nar* strains after several transfers in non-selective RCM and subsequent isolation of single colonies on non-selective RCM plates.

**Supplementary Text S1C - Growth of *C. ljungdahlii* WT with nitrate or ammonium as nitrogen source**

During autotrophy with ammonium, *C. ljungdahlii* WT showed a growth rate of 0.024±0.002 h^-1^, reached its maximum OD_600_ at 0.56±0.01, and produced 59.5±1.8 mM acetate and 1.9±0.4 mM ethanol (**Table 1, Figure 2**). When using nitrate instead, the growth rate was 0.072±0.004 h^-1^ and the maximum OD_600_ increased to 1.00±0.06 (**Table 1, Figure 2A**). This is a significant increase of 198% (*P* ≤ 0.001) and 79% (*P* ≤ 0.001) in comparison to ammonium conditions, respectively. In contrast, the maximum acetate concentration decreased to 50.1±2.1 mM, which is a significant reduction of 16% (*P* = 0.008), and maximum ethanol concentrations increased to 8.0±1.6 mM corresponding to a significant increase of 327% (*P* = 0.007) when using nitrate as nitrogen source (**Table 1, Figure 2C, 2D**). Notably, the ethanol production of nitrate grown cells only started after all nitrate was consumed at a cultivation time of 47 h. During heterotrophy, we observed that *C. ljungdahlii* WT performed slightly better in ammonium-containing medium in terms of growth and production of acetate and ethanol (**Supplementary Table S1, Supplementary Figure S1**). With nitrate and fructose, the growth rate decreased by 7% (*P* = 0.04), maximum OD_600_ values dropped by 10% (*P* = 0.03), and maximum concentrations for acetate were significantly reduced by 17% (*P* ≤ 0.001) and for ethanol by 53% (*P* ≤ 0.001) in comparison to growth with fructose and ammonium (**Supplementary Table S1**). During autotrophy, we observed that the pH of the medium initially increased up to pH 7.31, before it decreased again until the end of the cultivation (**Figure 1B**). The pH of ammonium cultures only decreased during the cultivation. This pH effect was not observed during heterotrophy, but the pH decreased slower for those cultures growing with nitrate (**Supplementary Figure S1B**). Notably, during heterotrophy, a halt in growth, pH decrease, and metabolic activity was observed after 55.5 h of cultivation when the fructose pool had been consumed completely (**Supplementary Figure S1**). In all our experiments, we found that a sufficient amount of nitrogen was supplied already by the yeast extract (1 g/L) that we had added, because further ammonium accumulated and was not consumed, even when only ammonium was provided as the nitrogen source. Similar observations were already reported by Emerson et al. (2019).

**Supplementary Text S1D – Effects of promoter strength and plasmid copy number on the plasmid-based complementation of the gene deletion strains**

Our complementation plasmids contained the Gram-positive origin of replication pCB102, which was originally derived from *Clostridium butyricum* (Heap et al., 2009). However, neither the exact replication mechanism nor the plasmid copy number are known. We speculated that at least several copies of our plasmid were present in our complementation strains. A negative impact on the growth due to a higher copy number of the *rnfCDGEAB* gene cluster in our complementation strain *C. ljungdahlii* pMTL83151_P_nat__*rnfCDGEAB* might explain the difference in maximum OD_600_ values and acetate concentrations (**Table 1, Supplementary Figure S3**). The difference between the *rnf*-complementation and the *rseC*-complementation strain might be explained by the different promoters that we used (non-characterized P_nat_ *vs.* constitutive P*_thl_*).

We observed a growth stimulating effect in the *C. ljungdahlii* ∆*rseC* pMTL83152_*rseC* strain, even though we typically find plasmid-carrying strains to perform worse, which we argued is due to the cellular burden that is generated by the expression of plasmid-encoded functions and the addition of antibiotics. To investigate whether overexpression of *rseC* in the wild-type strain increases autotrophic growth further, we generated the *C. ljungdahlii* pMTL83152_*rseC* strain. This strain carries the complementation plasmid with the constitutive P*_thl_* promoter in the wild-type background. During autotrophy with carbon dioxide and hydrogen in ammonium-containing medium of the *C. ljungdahlii* pMTL83152_*rseC* strain, the maximum acetate concentration was significantly reduced compared to the wild type and ethanol was not produced (**Supplementary Figure S4C, S4D**). Overall, this strain performed similar when compared to the *C. ljungdahlii* ∆*rseC* pMTL83152_*rseC* strain.

We also attempted to generate a plasmid that carries the *rnfCDGEAB* gene cluster under the control of a constitutive promoter. However, any attempts to generate a fusion of the constitutive promoter P*_thl_* with the *rnfCDGEAB* gene cluster failed already during the cloning steps in *E. coli*. Thus, for the expression of *rnfCDGEAB* in the wild type, we also used the native P_nat_ promoter sequence, which most likely is under the same expression control as the genomic copy of the RNF-gene cluster. Indeed, the cultivation of *C. ljungdahlii* pMTL83151_P_nat__*rnfCDGEAB* did not show any notable impact on growth and product formation when compared to the control strain that carried an empty plasmid, and the performance was similar to the complementation strain *C. ljungdahlii* ∆RNF pMTL83151_P_nat__*rnfCDGEAB* (**Supplementary Figure S4**).

**Supplementary Text S1E - The role of *rseC* genes in non-acetogens**

The *rseC* gene was already described as an important factor in the regulation of the oxidative stress response in *E. coli*, which is mediated by SoxR and the *rsxABCDGE* genes that share homology to the *rnf* genes in *R. capsulatus* (Koo et al., 2003). In *E. coli*, the *rseC* gene is organized in the *rseD*-*rpoE-rseABC* operon, but located separately from the *rsxABCDGE* genes (De Las Peñas et al., 1997; Missiakas et al., 1997; Koo et al., 2003). The proteins that are encoded by the *rseD*-*rpoE-rseABC* operon integrate signals from the redox state of SoxR, which senses the cellular levels of the oxidants superoxide and nitric oxide, and which is reduced by the membrane-bound complex Rsx. Thus, it is assumed that electrons from NADH are channeled through the Rsx complex and are transferred onto SoxR directly or indirectly to regenerate a reduced state after oxidation by oxidants (Koo et al., 2003; Biegel et al., 2011). The proposed system would be similar to the electron translocation of the RNF complex (Biegel et al., 2011). It is assumed that the RseC protein is responsible to regulate the expression of SoxR by repressing its own regulator gene *soxS* (Koo et al., 2003). In *E. coli,* a Tn*10*-transposon insertion mutant of *rseC* showed increased expression levels of the gene *soxS* (Koo et al., 2003). The level of *soxS* transcript was found to be responsible for the redox state of SoxR, and higher levels of *soxS* mRNA indicated higher oxidation rates of SoxR (Ding and Demple, 1997). Therefore, it is assumed that the function of RseC in *E. coli* is likely to keep the level of *soxS* transcript low, which then keeps SoxR in its reduced form (Koo et al., 2003). However, whether RseC interacts or interferes either with the Rsx complex or *soxS* transcript/SoxR is not understood, and neither is the function as transcription regulator (Koo et al., 2003). Koo et al. (2003) reported that *rseC* of *E. coli* shares homology to the N-terminal half of the *rnfF* gene in *R. capsulatus*, and thus postulated a regulatory function of *rseC* in the nitrogen fixation. In our *in-silico* analysis, we found that *rnfF* and *rseC* in *R. capsulatus* are likely separated genes (**Figure 4**). This does not exclude the potential role of *rseC* in the nitrogen fixation regulation, but it questions the homology between *rseC* and *rnf* genes. In addition, the *rnfF* gene is not part of the RNF complex gene cluster in genomes of acetogens.

**Supplementary Text S1F - Conservation of the RseC amino-acid sequence**

The conservation of the RseC amino-acid sequence was between 59% and 100% for *C. ljungdahlii*, *C. autoethanogenum*, *C. carboxidovorans,* and *C. kluyveri*, which is a high similarity (**Supplementary Figure S6**). In addition, the amino-acid sequence length is nearly identical with 138 amino acids (*C. ljungdahlii*, *C. autoethanogenum*, and *C. carboxidovorans*) and 137 amino acids (*C. kluyveri*), respectively. The second RseC homolog from *C. carboxidovorans* and *C. kluyveri* shared an identity of 65% with each other, but only between 25% and 49% to all other RseC proteins (**Supplementary Figure S6**). The RseC from *A. woodii* and *E. limosum* shared a similarity of 57% with each other, and only of 34% to 35% with the RseC proteins that are encoded directly upstream of the RNF-gene clusters (**Figure 4, Supplementary Figure S6**). The RseC proteins from *R. capsulatus* and *E. coli* have the same amino-acid sequence length (159 amino acids), but shared low similarities to each other (31%) as well as to the RseC proteins from the other microbes (18-34%) (**Supplementary Figure S6**). The similarity of the RseC protein from *C. ljungdahlii* and *R. capsulatus* was only 23%, while it was 36% for the RseC protein from *C. ljungdahlii* in comparison to the RseC protein from *E. coli*.


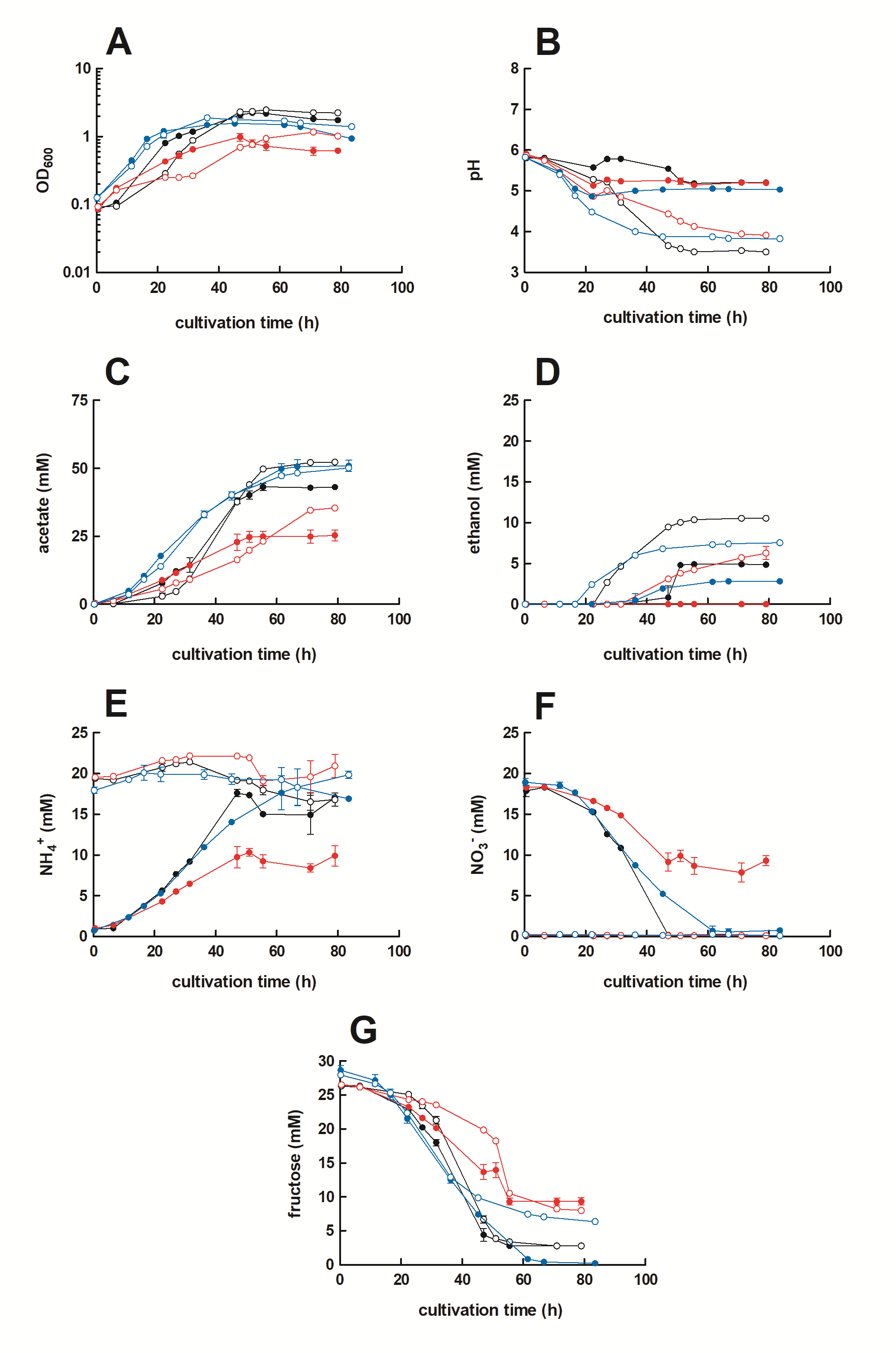


**Supplementary Figure S1.** **Heterotrophic growth and metabolic products of *C. ljungdahlii* WT, ΔRNF, and Δ*rseC*.** Cultures of *C. ljungdahlii* WT (●, ○), ΔRNF (●, ○), and Δ*rseC* (●, ○) were grown in 100 mL PETC medium in 240 mL bottles at 37°C. The headspace consisted of N_2_ (100 vol-%). Fructose (5 g/L) was added as carbon source. The medium contained either 18.7 mM nitrate (NO_3_^-^) (filled circles) or 18.7 mM ammonium (NH_4_^+^) (open circles) as nitrogen source. The cultivation times were 79 h for the WT and ΔRNF strain, and 84 h for the Δ*rseC* strain. All cultures were grown in biological triplicates, data is given as mean values, with error bars indicating the standard deviation. **A**, growth; **B**, pH-behavior; **C**, acetate concentrations; **D**, ethanol concentration; **E**, ammonium concentration; and **F**, nitrate concentrations. WT, wild type; ΔRNF, RNF-gene cluster deletion; Δ*rseC*, *rseC* gene deletion.


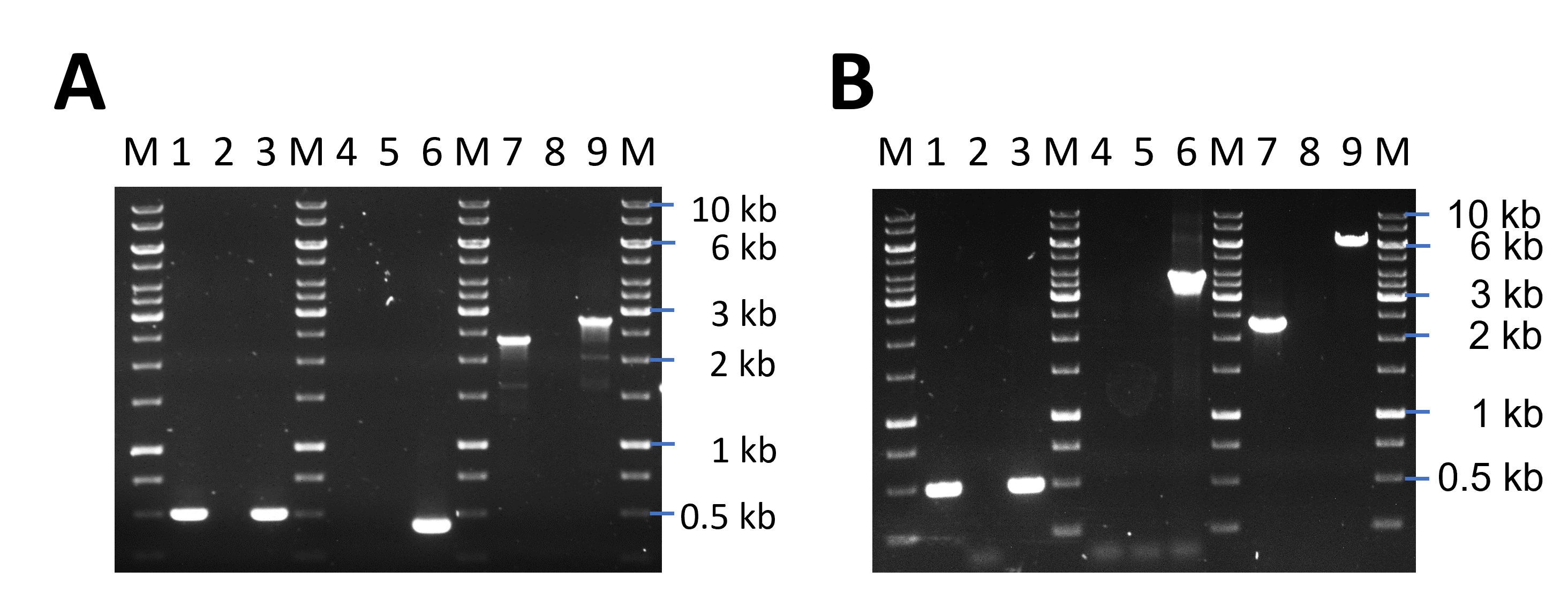


**Supplementary Figure S2**. **CRISPR-Cas12a-mediated *rseC* gene and *nar* gene cluster deletion in *C. ljungdahlii*.** **A**, verification of the *rseC* gene deletion. PCR-samples for the *fdhA* fragment (WT: 501 bp, deletion strain: 501 bp), *rseC* fragment (WT: 417 bp, deletion strain: no fragment), and for a fragment that was amplified with primers that bind 1104 bp upstream and 1208 bp downstream of the *rseC* gene locus (WT: 2755 bp, deletion strain: 2338 bp). DNA-template: gDNA of *C. ljungdahlii* Δ*rseC* (lane A1, A4, and A7); gDNA of *C. ljungdahlii* WT (lane A3, A6, and A9); and water (lane A2, A5, A8). **B**, verification of the *nar* gene cluster deletion PCR samples for the *fdhA* fragment (WT: 501 bp, deletion strain: 501 bp), *nar* fragment (WT: 3739 bp, deletion strain: no fragment), and for a fragment that was amplified with primers that bind 1137 bp upstream and 1110 bp downstream of the *nar* gene cluster locus (WT: 5986 bp, deletion strain: 2247 bp). DNA-template: gDNA of *C. ljungdahlii* Δ*nar* (lane B1, B4, and B7); gDNA of *C. ljungdahlii* WT (lane B3, B6, and B9); and water (lane B2, B5, B8). M: Generuler^TM^ 1 kb.


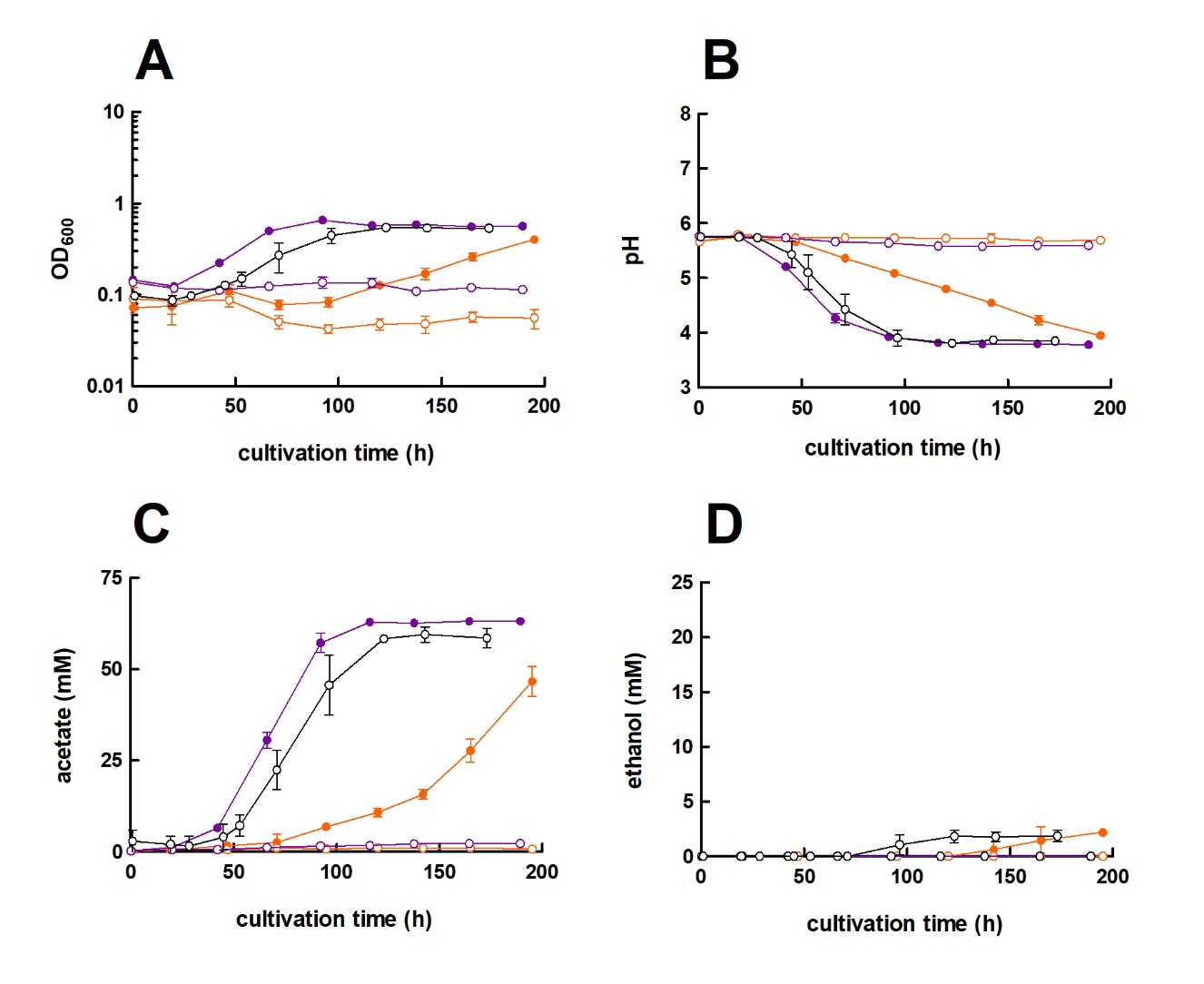


**Supplementary Figure S3. Growth and pH behavior of plasmid-based complementation of *C. ljungdahlii* ∆RNF and *C. ljungdahlii* ∆*rseC* with H_2_ and CO_2_.** Cultures were grown in 100 mL PETC medium in 1 L bottles at 37°C and 150 rpm for 195 h and 189 h, respectively. The headspace consisted of H_2_ and CO_2_ (80/20 vol-%) and was set to 0.5 bar overpressure. Only 18.7 mM ammonium (NH_4_^+^) but no nitrate was added to the medium. All cultures were grown in biological triplicates, data is given as mean values, with error bars indicating the standard deviation. The *C. ljungdahlii* WT data (○) from Fig. 1 is given for comparison. **A**, growth and **B**, pH-behavior, **C**, acetate concentration, and **D**, ethanol concentration of the *C. ljungdahlii* ΔRNF and Δ*rseC* strains. ● *C. ljungdahlii* ΔRNF pMTL83151_P_nat_*_rnfCDGEAB*; ○ *C. ljungdahlii* ΔRNF pMTL83151; ● *C. ljungdahlii* Δ*rseC* pMTL83152_*rseC*; and ○ *C. ljungdahlii* Δ*rseC* pMTL83152. ∆RNF, rnfCDGEAB gene cluster deletion; ∆*rseC*, deletion of *rseC*; P_nat_, native promoter sequence upstream of *rnfC*; P*_thl_*, promoter of the thiolase gene in *C. acetobutylicum*; rpm, revolutions per minute; CO_2_, carbon dioxide; and H_2_, hydrogen.


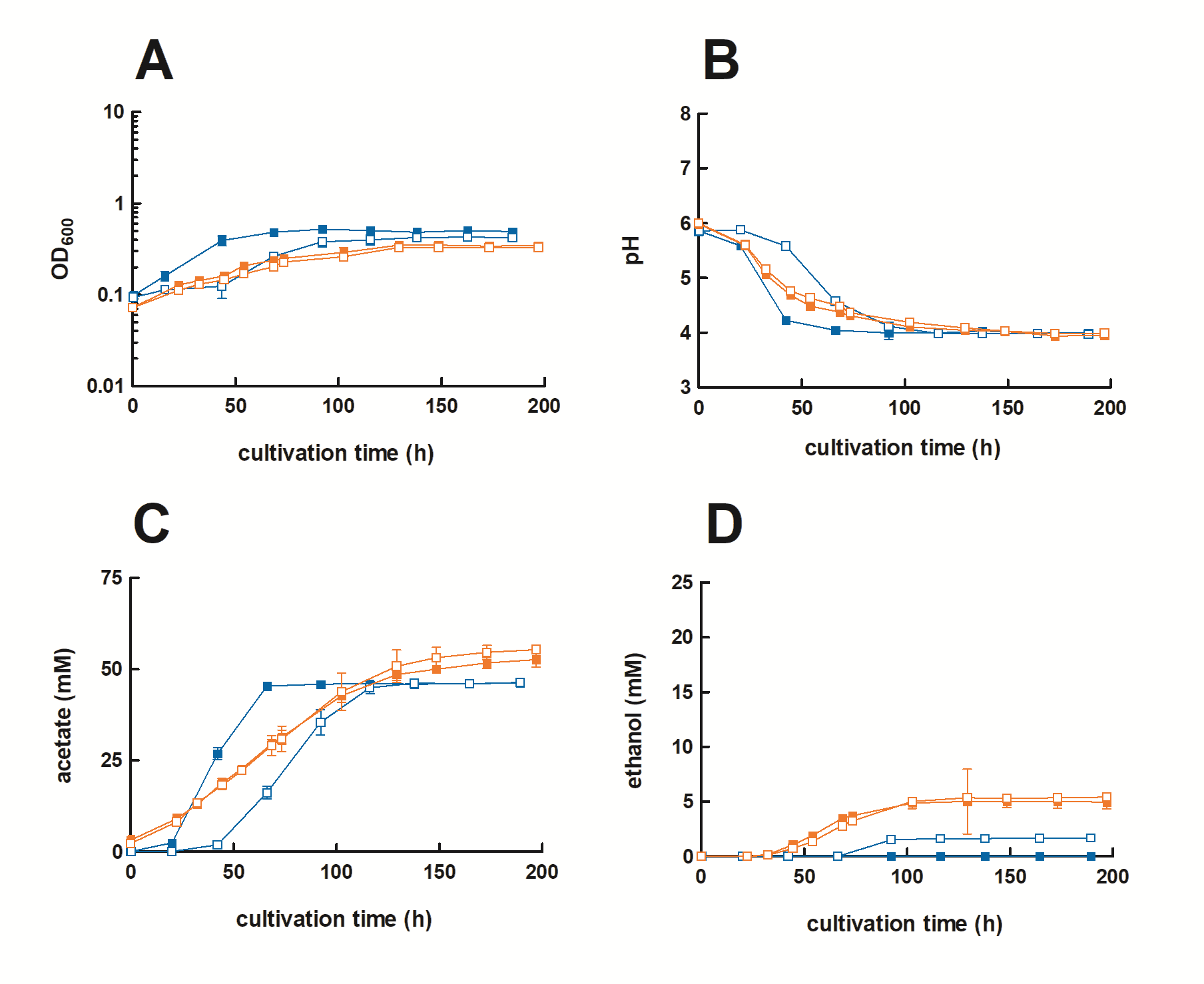


**Supplementary Figure S4. Autotrophic growth and metabolic products of the overexpression strains *C. ljungdahlii* pMTL83151_P_nat__*rnfCDGEAB* and *C. ljungdahlii* pMTL83152_*rseC*.** Cultures were grown in 100 mL PETC medium in 1 L bottles at 37°C and 150 rpm. The headspace consisted of H_2_ and CO_2_ (80/20 vol-%) and was set to 0.5 bar overpressure. For the strain *C. ljungdahlii* pMTL83151_P_nat__*rnfCDGEAB* and the control strain *C. ljungdahlii* pMTL83151 we refilled the headspace during this experiment with the same gas mixture to 0.5 bar overpressure at time points 44.5 h, 73.5 h, and 148.5 h. The medium contained 18.7 mM ammonium as nitrogen source. Thiamphenicol (5 µg/mL) was used for selection. All cultures were grown in biological triplicates, data is given as mean values, with error bars indicating the standard deviation. The cultivation time was 185 h and 197 h for *C. ljungdahlii* pMTL83151_P_nat__*rnfCDGEAB* and *C. ljungdahlii* pMTL83152_*rseC*, respectively. (■) *C. ljungdahlii* pMTL83151_P_nat__*rnfCDGEAB*; (□) *C. ljungdahlii* pMTL83151 (empty plasmid); (■) *C. ljungdahlii* pMTL83152_*rseC*; (□) *C. ljungdahlii* pMTL83152 (empty plasmid). The *C. ljungdahlii* WT data (○) from Figure 1 is given for comparison. **A**, growth; **B**, pH-behavior; **C**, acetate concentrations; and **D**, ethanol concentration. rpm, revolutions per minute; CO_2_, carbon dioxide; and H_2_, hydrogen.


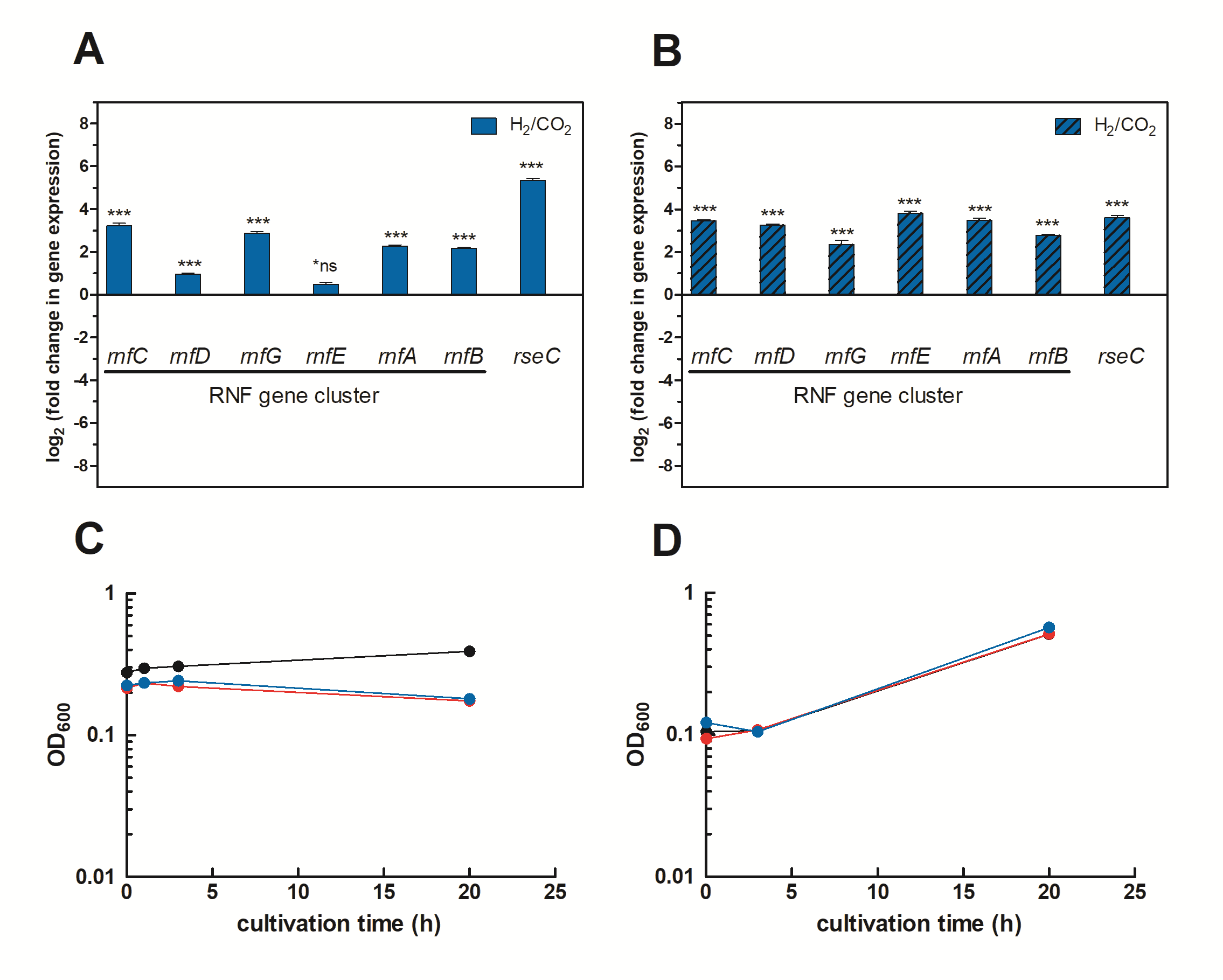


**Supplementary Figure S5. Gene expression change of the *rnfCDGEAB* cluster genes and the *rseC* gene in the wild-type strain from heterotrophy to autotrophy and growth curves of the cultivation experiments for the qPCR analyses. A**, gene expression change after 3 h cultivation time; **B**, gene expression change after 20 h cultivation time; **C**, growth of *C. ljungdahlii* WT (●), ΔRNF (●), and Δ*rseC* (●) during autotrophic conditions; **D,** growth of *C. ljungdahlii* WT (●), ΔRNF (●), and Δ*rseC* (●) during heterotrophic conditions. RNA samples were purified from cultures that were cultivated either autotrophically with hydrogen and carbon dioxide or heterotrophically with fructose. Cells of the deletion mutants *C. ljungdahlii* ΔRNF and *C. ljungdahlii* Δ*rseC* were not growing under autotrophic conditions and remained at their inoculation OD_600_ of 0.2±0.02 and 0.22±0.02, respectively. cDNA was synthesized from the purified RNA samples and used as template for qRT-PCR analyses. The *rho* gene was used as “housekeeping” gene. The fold change in gene expression was determined with the 2^-ΔΔCT^ method (Livak and Schmittgen, 2001). ***, *P* ≤ 0.001; and *ns, not significant (*P* > 0.5). We defined log_2_ (fc) ≤ -2 as downregulated genes and ≥ +2 as upregulated genes.


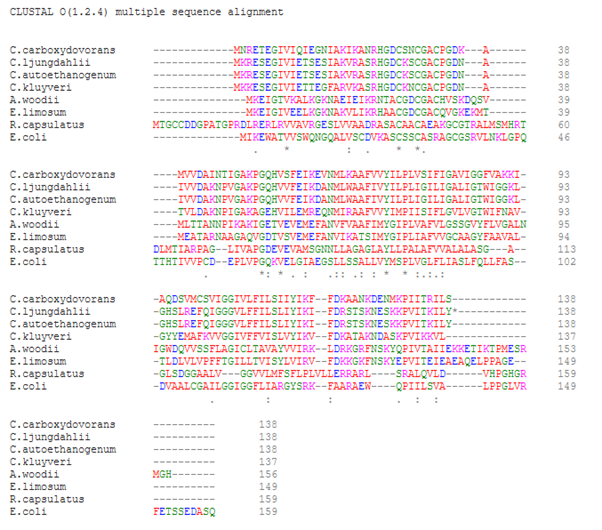


**Supplementary Figure S6. Multiple sequence alignment of RseC amino-acid sequence using CLUSTAL Omega.** The symbols indicate low similarity (.), high similarity (:), and identical amino acids (*) between the amino acid sequences. Similar colors indicate similar amino acids. The type strains were *C. ljungdahlii* DSM13528; *C. autoethanogenum* DSM10061; *C.  carboxidovorans* P7; *C. kluyveri* DSM555; *E. limosum* ATCC8486; *A. woodii* DSM1030; *R. capsulatus* SB1003; and *E. coli* K-12. Clustal omega version 1.2.4. with default settings was used for the analysis (https://www.ebi.ac.uk/Tools/msa/clustalo/, 05/2021). All listed RseC proteins are predicted to contain two transmembrane helices (https://services.healthtech.dtu.dk/service.php?TMHMM-2.0, (Möller et al., 2001)).


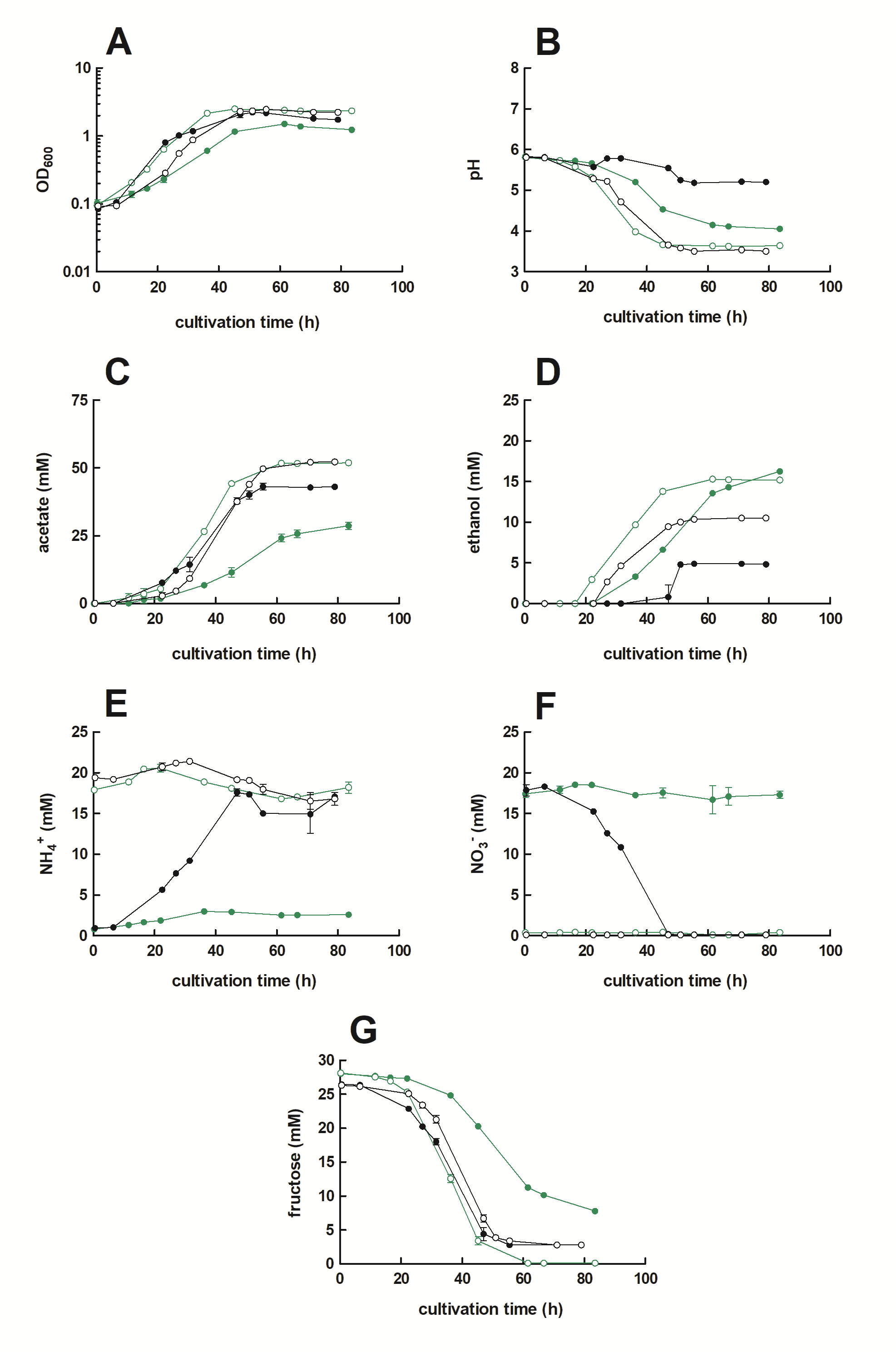


**Supplementary Figure S7. Heterotrophic growth and metabolic products of *C. ljungdahlii* ∆*nar*.** Cultures were grown in 100 mL PETC medium in 240 mL bottles at 37°C. Fructose (5 g/L) was added as carbon source. The headspace consisted of N_2_ (100 vol-%). The medium contained either 18.7 mM nitrate (●) or 18.7 mM ammonium (○) as nitrogen source. All cultures were grown in biological triplicates, data is given as mean values, with error bars indicating the standard deviation. The cultivation times was 84 h. The *C. ljungdahlii* WT data (●, ○) from Supplementary Figure S1 is given for comparison. **A**, growth; **B**, pH-behavior; **C**, acetate concentrations; **D**, ethanol concentration; **E**, ammonium concentration; **F**, nitrate concentrations; and **G**, fructose concentrations. ∆*nar*, deletion of the nitrate reductase genes.


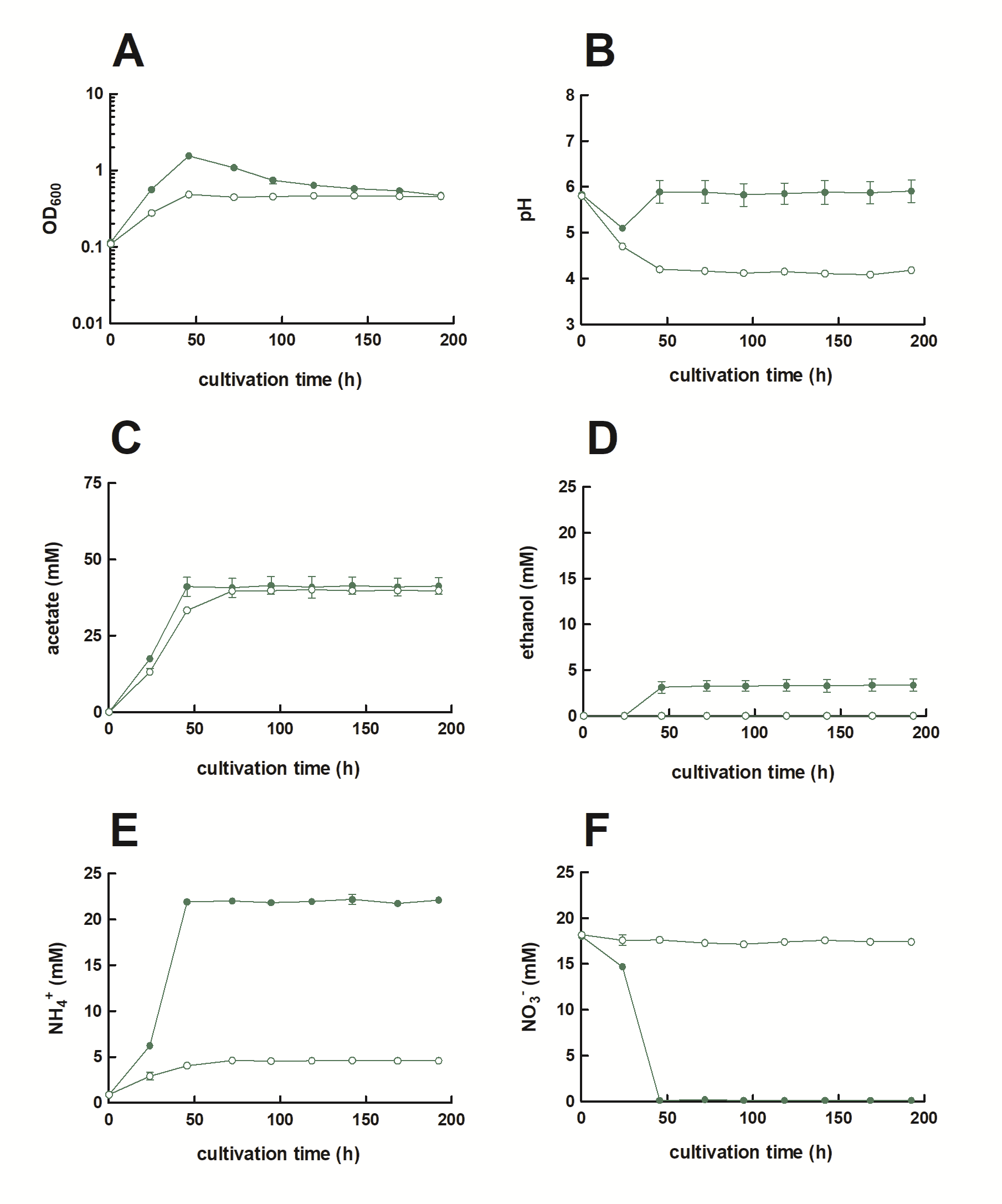


**Supplementary Figure S8. Autotrophic growth and metabolic products of plasmid-based complemented strain *C. ljungdahlii* ∆*nar* pMTL83152_*nar*.** Cultures were grown in 100 mL PETC medium in 1 L bottles at 37°C and 150 rpm. The headspace consisted of H_2_ and CO_2_ (80/20 vol-%) and was set to 0.5 bar overpressure. The medium contained 18.7 mM nitrate (NO_3_^-^) but no ammonium (NH_4_^+^) as nitrogen source. Thiamphenicol (5 µg/mL) was used for selection. All cultures were grown in biological triplicates, data is given as mean values, with error bars indicating the standard deviation. The cultivation times was 192.5 h. (●) *C. ljungdahlii* ∆*nar* pMTL83152_*nar*; (○) *C. ljungdahlii* ∆*nar* pMTL83152 (empty plasmid); **A**, growth, **B**, pH-behavior; **C**, acetate concentrations; **D**, ethanol concentration; **E**, ammonium concentration; and **F**, nitrate concentrations. ∆*nar*, gene deletion of the nitrate reductase genes; rpm, revolutions per minute; CO_2_, carbon dioxide; and H_2_, hydrogen.

Supplementary Table S1. Plasmids used in this study.

| **plasmid** | **function** | **source** |
| --- | --- | --- |
| pMTL83151 | shuttle-vector | (Heap et al., 2009) |
| pMTL83152 | shuttle-vector with constitutive thiolase promoter P*_th_*_l_ | (Heap et al., 2009) |
| pMTL2tetO1gusA | pMTL82254 with pminithl:tetR-O1 and  p2tetO1:gusA | (Woolston et al., 2018) |
| pMTL8315tet | shuttle-vector with inducible promoter system *tetR-O1* | this study |
| pMTL83151_P_nat__*rnfCDGEAB* | overexpression of *rnfCDGEAB* through native promoter P_nat_ | this study |
| pMTL83152_*rseC* | overexpression of *rseC* through constitutive promoter P*_thl_* | this study |
| pMTL83152_*nar* | overexpression of *nar* through constitutive promoter P*_thl_* | this study |
| pY001_FnCpf1(Cas12a) | expression of FnCas12a | (Zetsche et al., 2015), Addgene 69973 |
| pMTL83152_FnCas12a | constitutive expression of FnCas12a through P*_thl_* | this study |
| pMTL83152_FnCas12a_∆rseC | constitutive expression of FnCas12a through P*_thl_*, constitutive expression of a single sgRNA targeting *rseC* on the genome, fused repair HDR1/2 fragment for homologous recombination and marker-less gene deletion | this study |
| pMTL83152_FnCas12a_∆*nar* | constitutive expression of FnCas12a through P*_thl_*, constitutive expression of two sgRNA targeting *nar* on the genome, fused repair HDR1/2 fragment for homologous recombination and marker-less gene deletion | this study |
| pMTL83152_FnCas12a_∆*rnfCDGEAB* | constitutive expression of FnCas12a through P*_thl_*, constitutive expression of two sgRNA targeting *rnfCDGEAB* on the genome, fused repair HDR1/2 fragment for homologous recombination and marker-less gene deletion | this study |
| pMTL8315tet_FnCas12a | inducible expression of FnCas12a through tetR-O1 promoter system | this study |
| pMTL8315tet_FnCas12a_∆*rseC* | inducible expression of FnCas12a through tetR-O1 promoter system, constitutive expression of a single sgRNA targeting *rseC* on the genome, fused repair HDR1/2 fragment for homologous recombination and marker-less gene deletion | this study |
| pMTL8315tet_FnCas12a_∆*nar* | inducible expression of FnCas12a through tetR-O1 promoter system, constitutive expression of two sgRNA targeting *nar* on the genome, fused repair HDR1/2 fragment for homologous recombination and marker-less gene deletion | this study |
| pMTL8315te_FnCas12a_∆*rnfCDGEAB* | inducible expression of FnCas12a through tetR-O1 promoter system, constitutive expression of two sgRNA targeting *rnfCDGEAB* on the genome, fused repair HDR1/2 fragment for homologous recombination and marker-less gene deletion | this study |

Supplementary Table S2. Primers used in this study.

| **primer** | **sequence (5’-> 3’)** | **function** |
| --- | --- | --- |
| rnfCDGEAB+213bp_fw_BamHI | GGATCCGTAATTTGTGTACAAACTTTAATTAATGGAGAGAC | amplification of the RNF complex gene cluster (CLJU_c11360-410) + and promoter sequence (P_nat_) |
| rnfCDGEAB_rv_NcoI | CCATGGTTATGAATTTGCAGCAGCTTCATTCTTG | amplification of the RNF complex gene cluster (CLJU_c11360-410) + and promoter sequence (P_nat_) |
| rseC_fwd_BamHI | GGATCCAGGAGGTTAAGAATGAAAAGAGAATCGGAGGGTATTG | amplification of the putative RNF regulator gene *rseC* (CLJU_c11350) |
| rseC_rv_NcoI | CCATGGTCAATACAATATCTTTGTGATTACTGGC | amplification of the putative RNF regulator gene *rseC* (CLJU_c11350) |
| nar-full_fwd_BamHI | GGCAGCTTACCGGGATCCAGGAGGTTAAGAATGAATTACGTGGAAGTAAAACAATCAAC | amplification of a gene cluster (CLJU_c23710-30) encoding for a nitrate reductase |
| nar-full_rv_NcoI | GCACGGTCGTCGCCATGGTTAAAAAGTATACTCTAAATTTTCCTTTATATTAAAAAAGTC | amplification of a gene cluster (CLJU_c23710-30) encoding for a nitrate reductase |
| Seq1_RNF_744bp_fwd | GGAAAATTCAGACAAGGTAGTTGC | sanger sequencing of the *rnfCDGEAB* fragment |
| Seq2_RNF _1502bp_fwd | CAGAAAATAGAGCTGCAGGTGAAAG | sanger sequencing of the *rnfCDGEAB* fragment |
| Seq3_RNF _2268bp_fwd | CTGGCAGATTCCAGTAGTAATGATTG | sanger sequencing of the *rnfCDGEAB* fragment |
| Seq4_RNF _3000bp_fwd | GGGACAGTTTAAGGATAAAAAGGCAG | sanger sequencing of the *rnfCDGEAB* fragment |
| Seq5_RNF_3787bp_fwd | GCAAATGGAGGTGAAGCATAATG | sanger sequencing of the *rnfCDGEAB* fragment |
| Seq6_RNF _4502bp_fwd | GTGAATCCACTTGTAGACTTAGTAGAAG | sanger sequencing of the *rnfCDGEAB* fragment |
| Seq7_RNF_5047bp_rv | TTATGAATTTGCAGCAGCTTCATTCTTG | sanger sequencing of the *rnfCDGEAB* fragment |
| Seq1 _nar_456bp_rv | GCACCTCCTTATACTCTAAAAGATTTTG | sanger sequencing of the *nar* fragment |
| Seq2 _nar_610bp_fwd | CTGTTTCAGATTTTCTCGGGTCAATTG | sanger sequencing of the *nar* fragment |
| Seq3 _nar_1059bp_rv | CCAAAGCATAGAGAAGAAATTGC | sanger sequencing of the *nar* fragment |
| Seq4 _nar_1186bp_fwd | CCCACAATGCCTTAATTTCTCCG | sanger sequencing of the *nar* fragment |
| Seq5 _nar_1677bp_rv | GTAAAGCTCATTTATGAAGATGCAGCC | sanger sequencing of the *nar* fragment |
| Seq6 _nar_1798bp_fwd | CCCTAGTTCTAGTCTGGGTATGC | sanger sequencing of the *nar* fragment |
| Seq7 _nar_2303bp_rv | CCAGATACCGGTATTGTAGAGTACG | sanger sequencing of the *nar* fragment |
| Seq8 _nar_2476bp_fwd | CATCTAGCTACACACTGCGG | sanger sequencing of the *nar* fragment |
| Seq9 _nar_2902bp_rv | GATGCACAAAAAATAAAGGATGCAGC | sanger sequencing of the *nar* fragment |
| Seq10 _nar_3086bp_fwd | CTTCATATCTGCCTGCTGCA | sanger sequencing of the *nar* fragment |
| Seq11 _nar_3385bp_rv | GGAATTGTAGCAGCTAGTAATATGGC | sanger sequencing of the *nar* fragment |
| tetR-O1_fwd_SbfI | CCTGCAGGATAAAAAAATTGTAGATAAATTTTATAAAATAG | amplification of the inducible promoter system tetR-O1 |
| tetR-O1_rv_BamHI | GGATCCTATTTCAAATTCAAGTTTATCGCTCTAATGAAC | amplification of the inducible promoter system tetR-O1 |
| repH _401bp_rv | CTCTAACGGCTTGATGTGTTGG | primer binding in the backbone of pMTL83151 and pMTL83152 upstream of *repH* |
| fdhA_fwd | AGTGCAGCGTATTCGTAAGG | amplification of a 501 bp fragment of the *fdhA* gene in *C. ljungdahlii* |
| fdhA_rv | TAATGAGCCACGTCGTGTTG | amplification of a 501 bp fragment of the *fdhA* gene in *C. ljungdahlii* |
| repH_643bp_rv | GCACTGTTATGCCTTTTGACTATCAC | primer binding in the backbone of pMTL83151 and pMTL83152 upstream of *repH* |
| traJ_60bp_fw | CATGCGCTCCATCAAGAAGAG | primer binding in the backbone of pMTL83151 and pMTL83152 downstream of *traJ* |
| rnfC_250bp_rv | CTCCTATATCTACAACCTTTCCAGAAGTAG | primer binding 250 bp upstream of *rnfC*, which was used for sanger sequencing and PCR screening |
| cas12a_fwd_BamHI | GGTACCGGATCCATGTCAATTTATCAAGAATTTGTTAATA | amplification of *Fncas12a* |
| cas12a_rv_NcoI | GGTACCCCATGGTTAGTTATTCCTATTCTGCAC | amplification of *Fncas12a* |
| Seq1_cas12a | CACAGATATAGATGAGGCG | sanger sequencing of *Fncas12a* |
| Seq2_cas12a | GCTTCTGGAGCTTTGTCT | sanger sequencing of *Fncas12a* |
| Seq3_cas12a | GTAGTTACAACGATGCAAAG | sanger sequencing of *Fncas12a* |
| Seq4_cas12a | CCGCTGTACCAATAACAC | sanger sequencing of *Fncas12a* |
| Seq5_cas12a | GGCTAATGGTTGGGATAA | sanger sequencing of *Fncas12a* |
| Seq6_cas12a | CTTATTCATCACACCCAG | sanger sequencing of *Fncas12a* |
| Seq7_cas12a | CAAGATGTGGTTTATAAGC | sanger sequencing of *Fncas12a* |
| Seq8_cas12a | CCTCTTTAGCTGGGTGAGTG | sanger sequencing of *Fncas12a* |
| Seq9_cas12a | CAAGGTAGAGAAGCAGGTC | sanger sequencing of *Fncas12a* |
| Seq10_cas12a | GCTCTAAGCACTCCCCCAG | sanger sequencing of *Fncas12a* |
| Seq11_cas12a | GCTAAGCTAACTAGTGTC | sanger sequencing of *Fncas12a* |
| Seq12_cas12a | CCATTTACATCTGCTACTGG | sanger sequencing of *Fncas12a* |
| HDR_rnfB_fwdOv | TGTAAAAATTATTGAAAGAGGTGTTTAAGATGGCAGTGGAGCAAAGCTT | amplification of homology-directed repair arm downstream of *rnfB* with overhang to the homology-directed repair arm upstream of *rnfC* |
| HDR_rnfB_rv | ATGTAAAGGGTTCACATAAAATAGCTGT | amplification of homology-directed repair arm downstream of *rnfB* |
| HDR_rnfC_fwdOv | CAAGTTGAAAAATTTAATAAAAAAATAAGTGGCTTGAAATCAATAGTTAACGCAATAG | amplification of homology-directed repair arm upstream of *rnfC* with overhang to the *Fncas12a* sequence |
| HDR_rnfC_fwd | GGCTTGAAATCAATAGTTAACGCAATAG | amplification of homology-directed repair arm upstream of *rnfC* without overhang |
| HDR_rnfC_rvOV | TCAGCAAATTTAAGCTTTGCTCCACTGCCATCTTAAACACCTCTTTCAATAATTTTTACAGC | amplification of homology-directed repair arm upstream of *rnfC* with overhang to the homology-directed repair arm downstream of *rnfB* |
| Seq_HDR_rnfB_881bp_fwd | GACCTGGTTCGGATATCCATCC | sanger sequencing of the HDR_rnfB fragment |
| minigene_crRNA_RNF_fwd | TTTATGTGAACCCTTTACATTTGACAAATT | amplification of crRNA array consisting of 22-bp overhang to HDR_rnfB , p4-promoter, direct repeats, sgRNA (TTA), and rrnB-T1 terminator for genome target *rnfCDGEAB* |
| minigene_crRNA_all_rv | GTTGGTAGCTTAATATATAAGAATAAAACGAAAGG | amplification of crRNA array consisting of 22-bp overhang to pMTL83152-Cas12a, p4-promoter, direct repeats, sgRNA (TTA), and rrnB-T1 terminator for genome target *rnfCDGEAB, rseC,* and *nar* |
| outside_RNF_HDR_dwst_rv | GCATGGGAGTGTTAATATGAAAAAAGGG | verification of *rnfCDGEAB* deletion |
| outside_RNF_HDR_upst_fwd | GGAGGCTATTAAGGGACCGT | verification of *rnfCDGEAB* deletion |
| HDR_rseC_dwst_fwdOv | CGCTAACAAATAATAGGAGGTGTATTATGTAATTTGTGTACAAACTTTAATTAATGGAGAGAC | amplification of a homology-directed repair arm downstream of *rseC* with 28-bp overlap to HDR_rseC_upst |
| HDR_rseC_dwst_rv | TAGTTGTAACCCTCTGTATAAGTGGAATTC | amplification of a homology-directed repair arm downstream of *rseC* |
| HDR_rseC_upst_fwd | CTCATTGAAGTATATGTTAATGGCAGAAAAAAAGTTC | amplification of a homology-directed repair arm upstream of *rseC* |
| HDR_rseC_upst_fwdOv | CAAGTTGAAAAATTTAATAAAAAAATAAGTCTCATTGAAGTATATGTTAATGGCAGAAAAAAAGTTC | amplification of a homology-directed repair arm upstream of *rseC* with 30-bp overlap to pMTL83152-Cas12a |
| HDR_rseC_upst_rvOv | TCCATTAATTAAAGTTTGTACACAAATTACATAATACACCTCCTATTATTTGTTAGCGTTTTC | amplification of a homology-directed repair arm upstream of *rseC* with 30-bp overlap to fragment HDR_rseC_dwst |
| minigene_crRNA_rseC_fwd | CTTATACAGAGGGTTACAACTATTGACAAATT | amplification of crRNA array consisting of 22-bp overhang to HDR_rseC_dwst, p4-promoter, direct repeats, sgRNA (TTA), and rrnB-T1 terminator for genome target *rseC* |
| outside_rseC_HDRdwst_rv | CCCATCATAGGTCCACCTGAAA | verification of *rseC* deletion |
| outside_rseC_HDRupst_fwd | CGAGCTGAAGGTTGTAAAAATATCCG | verification of *rseC* deletion |
| seq_rseC_145bpupst_fwd | GAAGGTAATACTGTTCAATATCGATACAGA | verification of *rseC* deletion |
| HDR_nar_dwst_fwdOv | TCTTTTTCATAAATTTAGAGTATACTTTCTCCACTTCTCAATATTTTTTACTGAAAATAC | amplification of a homology-directed repair arm downstream of nar with overhang to HDR_nar_upst |
| HDR_nar_dwst_rv | TTGGAATGACAGGACTCTATATAGTTATGG | amplification of a homology-directed repair arm downstream |
| HDR_nar_upst_fwd | TACAACCTCTGTTAGTACTGCTGATATTACATC | amplification of a homology-directed repair arm upstream of nar |
| HDR_nar_upst_fwdOv | CAAGTTGAAAAATTTAATAAAAAAATAAGTTACAACCTCTGTTAGTACTGCTGATATTACATC | amplification of a homology-directed repair arm upstream of nar with overhang to *Fncas12a* |
| HDR_nar_upst_rvOv | GTATTTTCAGTAAAAAATATTGAGAAGTGGAGAAAGTATACTCTAAATTTATGAAAAAGAATTTTA | amplification of a homology-directed repair arm upstream of *nar* with overhang to HDR_nar_dwst |
| minigene_nar_fwd | ATATAGAGTCCTGTCATTCCAATTGACAAATT | amplification of crRNA array consisting of 22-bp overhang to HDR_nar_dwst, p4-promoter, direct repeats, sgRNA (TTA), and rrnB-T1 terminator for genome target *nar* |
| seq_nar_95bp_dwst_fwd | CCGGATAACCTTTAGTGGGAAGT | verification of *nar* deletion |
| seq_nar_132bp_upst_rv | GCGCCATAATTCAAGGGGAT | verification of *nar* deletion |
| outside_nar_HDRdwst_rv | GGGTTGACGTAGATGGAGGAAG | verification of *nar* deletion |
| outside_nar_HDRupst_fwd | CCTTTAAGCTTCCACCATTTGCC | verification of *nar* deletion |
| qPCR_rseC_fwd | GCTAGTAGACACGGAGATTG | amplification of a 142 bp fragment from *rseC* |
| qPCR_rseC_rv | CTGCCCATAACATATTTGC | amplification of a 142 bp fragment from *rseC* |
| qPCR_rnfC_fwd | GCACCTATACCAGATAAGGT | amplification of a 160 bp fragment from *rnfC* |
| qPCR_rnfC_rv | CCTTTCCAGAAGTAGATGCAT | amplification of a 160 bp fragment from *rnfC* |
| qPCR_rnfD_fwd | CCTCATGTTCGTTGTGATG | amplification of a 157 bp fragment from *rnfD* |
| qPCR_rnfD_rv | CAAAGTACTCCGTAACTACAGC | amplification of a 157 bp fragment from *rnfD* |
| qPCR_rnfG_fwd | CATCACCAGTAGCAGCG | amplification of a 156 bp fragment from *rnfG* |
| qPCR_rnfG_rv | CTGCAGGTACAACATATGC | amplification of a 156 bp fragment from *rnfG* |
| qPCR_rnfE_fwd | TGTGTCCAGCACTGGC | amplification of a 138 bp fragment from *rnfE* |
| qPCR_rnfE_rv | CAGGGACACGTACCTTAG | amplification of a 138 bp fragment from *rnfE* |
| qPCR_rnfA_fwd | GCATCTGTAGGTATGGGTATG | amplification of a 136 bp fragment from *rnfA* |
| qPCR_rnfA_rv | CAATAAGAAGTACAAAAACTACCG | amplification of a 136 bp fragment from *rnfA* |
| qPCR_rnfB_fwd | GCAATGGAAGTGAATCCAC | amplification of a 155 bp fragment from *rnfB* |
| qPCR_rnfB_rv | GCTGCTTTTCCAGGTAC | amplification of a 155 bp fragment from *rnfB* |
| qPCR_rho_fwd | GGACTCTTTCAGGAGGACTA | amplification of a 243 bp fragment from *rho* |
| qPCR_rho_rv | ATACATCTATGGCAGGGAAT | amplification of a 243 bp fragment from *rho* |

Supplementary Table S3. Synthesized mini genes that contain crRNA arrays for this study. Gene synthesis was performed by IDT (Integrated DNA Technologies). Each mini gene contains 20-22-bp overhang to the pMTL-backbone and to the fused homology-directed repair arms. Directed-repeats sequence of 20 bp (underlined). sgRNA with TTV PAM for the RNF complex gene cluster deletion and with TTTV PAM for the *nar* and *rseC* deletion (bold). Two sgRNAs were used to target RNF and *nar*.

| **name** | **sequence (3’-> 5’)** |  |
| --- | --- | --- |
| minigene_crRNA-RNF | TTTATGTGAACCCTTTACATTTGACAAATTTATTTTTTAAAGTTAAAATTAAGTTGTAATTTCTACTGTTGTAGAT**AAAAGTTTTCGAGGTGGAGTACA**TAATTTCTACTGTTGTAGAT**CAACAGCAGAGCAAGAATGAAGC**ATAAAACGAAAGGCTCAGTCGAAAGACTGGGCCTTTCGTTTTATTCTTATATATTAAGCTACCAAC |  |
| minigene_crRNA-*rseC* | CTTATACAGAGGGTTACAACTATTGACAAATTTATTTTTTAAAGTTAAAATTAAGTTGTAATTTCTACTGTTGTAGAT**ATAGATCTACAAGCAAAAATGAG**ATAAAACGAAAGGCTCAGTCGAAAGACTGGGCCTTTCGTTTTATTCTTATATATTAAGCTACCAAC |  |
| minigene_crRNA-*nar* | ATATAGAGTCCTGTCATTCCAATTGACAAATTTATTTTTTAAAGTTAAAATTAAGTTGTAATTTCTACTGTTGTAGAT**TATTTCTTGTTTATAGCTTTCAT**TAATTTCTACTGTTGTAGAT**TACAGCAAAATCCATCATTTACC**ATAAAACGAAAGGCTCAGTCGAAAGACTGGGCCTTTCGTTTTATTCTTATATATTAAGCTACCAAC |  |
|  |  |  |

Supplementary Table S4. Used and generated *C. ljungdahlii* strains in this study.

| **clostridial strain** | **plasmid** | **phenotype** | |
| --- | --- | --- | --- |
|  |  | **heterotrophic** | **autotrophic** |
| *C. ljungdahlii* DSM13528 | - | yes | yes |
| *C. ljungdahlii* DSM13528 | pMTL83151 | yes | yes |
| *C. ljungdahlii* DSM13528 | pMTL83151_P*_tetR-O1_* | yes | yes |
| *C. ljungdahlii* DSM13528 | pMTL83152 | yes | yes |
| *C. ljungdahlii* DSM13528 | pMTL83151_P_nat__*rnfCDGEAB* | yes | yes |
| *C. ljungdahlii* DSM13528 | pMTL83152_*rseC* | yes | yes |
| *C. ljungdahlii* DSM13528 | pMTL83151_*nar* | yes | yes |
| *C. ljungdahlii* ∆RNF**^a^** | - | yes (reduced) | no |
| *C. ljungdahlii* ∆RNF**^a^** | pMTL83151 | yes (reduced) | no |
| *C. ljungdahlii* ∆RNF**^a^** | pMTL83151_P_nat__*rnfCDGEAB* | yes | yes |
| *C. ljungdahlii ∆rseC* | - | yes | no |
| *C. ljungdahlii ∆rseC* | pMTL83152 | yes | no |
| *C. ljungdahlii ∆rseC* | pMTL83152 _*rseC* | yes | yes |
| *C. ljungdahlii ∆nar* | - | yes | yes |
| *C. ljungdahlii ∆nar* | pMTL83152 | yes | yes |
| *C. ljungdahlii ∆nar* | pMTL83152_*nar* | yes | yes |

**^a^** ∆RNF = ∆*rnfCDGEAB*

Supplementary Table S5. Performance of all tested *C. ljungdahlii* strains in heterotrophic batch cultivation experiments. Cultures were grown with fructose (5 g/L) in PETC medium, which contained either ammonium or nitrate as nitrogen source. All growth experiments were performed under anaerobic conditions. Data is given as mean values ± standard deviation from biological triplicates. WT, *C. ljungdahlii* wild type; ∆RNF, *C. ljungdahlii* with deleted *rnfCDGEAB* gene cluster; ∆*rseC*, *C. ljungdahlii* with deleted *rseC* gene; and ∆*nar,* *C. ljungdahlii* with deleted nitrate reductase gene cluster. Given in percentage is the difference in performance in comparison to the wild type with the same nitrogen source.

| **strain** | **nitrogen source** | **growth rate (µ in h)^a^** | **maximum OD_600_ value** | **maximum acetate concentration (mM)** | **maximum ethanol concentration (mM)** |
| --- | --- | --- | --- | --- | --- |
| WT | ammonium | 0.079±0.002 | 2.49±0.03 | 52.3±0.7 | 10.6±0.1 |
| WT | nitrate | 0.073±0.002 | 2.24±0.10 | 43.6±0.6 | 5.0±0.1 |
| ΔRNF | ammonium | 0.052±0.003  (-34%, ***) | 1.16±0.03  (-53%, ***) | 35.4±0.5  (-32%, ***) | 6.3±0.8  (-32%, n.s.^c^) |
| ΔRNF | nitrate | 0.042±0.003  (-42%, ***) | 0.98±0.10  (-56%, ***) | 25.4±1.7  (-42%, ***) | n.d.**^b^** |
| Δ*rseC* | ammonium | 0.084±0.002  (6%, n.s.^c^) | 1.90±0.15  (-31%, ***) | 50.1±0.3  (-4%, n.s.^c^) | 7.5±0.1  (-29%, ***) |
| Δ*rseC* | nitrate | 0.048±0.002  (-34%, ***) | 1.58±0.03  (-30%, ***) | 50.9±1.7  (-3%, n.s.^c^) | 2.9±0.1  (-42%, ***) |
| Δ*nar* | ammonium | 0.071±0.002  (-11%, **) | 2.35±0.04  (-6%, n.s.^c^) | 51.9±0.9  (-1%, n.s.^c^) | 15.3±0.1  (+44%, ***) |
| Δ*nar* | nitrate | 0.067±0.001  (-9%, n.s.^c^) | 1.51±0.03  (-32%, ***) | 28.7±1.1  (-34%, ***) | 16.6±0.2  (+234%, ***) |

**^a^** µ values were calculated based on the individual OD_600_ values of each triplicate in the exponential growth phase.

**^b^** n.d., not detectable.

^c^ n.s., not significant (*P* > 0.05)

*, significant (*P* ≤ 0.05)

**, significant (*P* ≤ 0.01)

***, significant (*P* ≤ 0.001)

Supplementary Table S6. RseC peptide sequences and amount of predicted transmembrane helices. Putative transmembrane helices were predicted with the TMHMM-2.0 tool (<https://services.healthtech.dtu.dk/service.php?TMHMM-2.0>) based on the Rsec peptide sequence.

| microbe | RseC peptide sequence | predicted transmembrane helices |
| --- | --- | --- |
| *C. ljungdahlii* | MKRESEGIVIETSESIAKVRASRHGDCKSCGACPGDNAIVVDAKNPVGAKPGQHVVFEIKDANMLWAAFIVYILPLIGILIGALIGTWIGGKLGHSLREFQIGGGVLFFILSLIYIKIFDRSTSKNESKKPVITKILY | 2 |
| *C. autoethanogenum* | MKRESEGIVIETSESIAKVRASRHGDCKSCGACPGDNAIVVDAKNPVGAKPGQHVVFEIKDANMLWAAFIVYILPLIGILIGALIGTWIGGKLGHSLREFQIGGGVLFFILSLIYIKIFDRSTSKNESKKPVITKILY | 2 |
| *C. carboxidovorans* | MNRETEGIVIQIEGNIAKIKANRHGDCSNCGACPGDKAMVVDAINTIGAKPGQHVSFEIKEVNMLKAAFVVYILPLVSIFIGAVIGGFVAKKIAQDSVMCSVIGGIVLFILSIIYIKFFDKAANKDENMKPIITRILS | 2 |
| *C. kluyveri* | MKKESEGIVIETTEGFARVKASRHGDCKNCGACPGDNATVLDAKNPIGAKAGEHVILEMREQNMIRAAFVVYIMPIISIFLGVLVGTWIFNAVGYYEMAFKVVGGIVFFVISLVYIKVFDKATAKNDASKPVIKKVL | 2 |
| *E. limosum* | MKEIGIVEELKGKNAKVLIKRHAACGDCGACQVGKEKMTMEATARNAAGAQVGDTVSVEMEFANVIKATSIMYGIPLIAFVVGCAAGYFAAVALTLDLVLVPFFTGILLTVISYLVIRVFDKKGKFNSKYEPVITEIEAEAQELPPAGE | 2 |
| *A. woodii* | MKEIGTVKALKGKNAEIEIKRNTACGDCGACHVSKDQSVMLTTANNPIKAKIGETVEVEMEFANVFVAAFIMYGIPLVAFVLGSSGVYFLVGALNIGWDQVVSSFLAGICLTAVAYVVIRKLDRKGRFNSKYQPIVTAIIEKKETIKTPMESRMGH | 2 |
| *R. capsulatus* | MTGCCDDGPATGPRDLRERLRVVAVRGESLVVAADRASACAACAEAKGCGTRALMSMHRTDLMTIARPAGLIVAPGDEVEVAMSGNNLLAGAGLAYLLPALAFVVALALASGAGLSDGGAALVGGVVLMFSFLPLVLLERRARLSRALQVLDVHPGHGR | 2 |
| *E. coli* | MIKEWATVVSWQNGQALVSCDVKASCSSCASRAGCGSRVLNKLGPQTTHTIVVPCDEPLVPGQKVELGIAEGSLLSSALLVYMSPLVGLFLIASLFQLLFASDVAALCGAILGGIGGFLIARGYSRKFAARAEWQPIILSVALPPGLVRFETSSEDASQ | 2 |

Supplementary Table S7. Distribution of potential *rseC* genes in genomes of acetogens. Given the availability of full genomes in NCBI, 47 of the 61 acetogenic bacteria listed in Table 2 from Bengelsdorf et al. (2018) were considered in this analysis. Using the NCBI Datasets API, ncbi-datasets-pylib 12.15.0, the GenBank files of these 47 acetogens were retrieved and used to generate protein FASTA files. Using BLASTp (BioPython 1.79), the *rseC* and RNF-gene cluster from *Clostridium ljungdahlii* DSM 13528 (CLJU_c11350- CLJU_c11410), and four subunits from the Ech-gene cluster, *ech2A1, ech2A2, ech2B, echE2,* from *Thermoanaerobacter kivui* (TKV_c19720, TKV_c19710, TKV_c19690, TKV_C19740) were used as the queries to the 47 genomes. An expect value (E-value) of 10e^-10^ was used as a threshold for finding potential gene matches (<https://resources.qiagenbioinformatics.com/manuals/clcgenomicsworkbench/650/_E_value.html>). Abbreviations: Y, yes, present; N, no, not present; and ID, identity.

| **Acetogenic bacterium^a^** | **Assembly ID** | ***rseC* gene ID^b^** | ***rnfC* gene ID** | ***rnf*-gene cluster (ABCDEG)^c^** | ***rseC* gene flanking *rnf*C gene^d^** | ***ech2A1* gene ID^e^** | ***ech2A2* gene ID^e^** | ***ech2B* gene ID^e^** | ***echE2* gene ID^e^** |
| --- | --- | --- | --- | --- | --- | --- | --- | --- | --- |
| *Acetitomaculum ruminis* DSM 5522 | GCF_900112085.1 | --- | ['BM153_RS11735'] | ['Y'] | --- | --- | --- | --- | --- |
| *Acetoanaerobium noterae* | GCF_900168025.1 | ['B5X47_RS10270', 'B5X47_RS12780'] | ['B5X47_RS09760'] | ['Y'] | [['N'], ['N']] | ['B5X47_RS08535'] | ['B5X47_RS08535'] | --- | --- |
| *Acetobacterium bakii* | GCF_900235925.1 | ['DXY11_RS15255'] | ['DXY11_RS15080'] | ['Y'] | [['N']] | ['DXY11_RS11030', 'DXY11_RS11045'] | ['DXY11_RS11045', 'DXY11_RS11030'] | --- | ['DXY11_RS11050'] |
| *Acetobacterium dehalogenans* DSM 11527 | GCF_000472665.1 | ['A3KS_RS0106250', 'A3KS_RS0116425', 'A3KS_RS0114380'] | ['A3KS_RS0106245', 'A3KS_RS0114035'] | ['Y', 'Y'] | [['Y', 'N'], ['N', 'N'], ['N', 'N']] | ['A3KS_RS0109555'] | ['A3KS_RS0109555'] | --- | --- |
| *Acetobacterium fimetarium* | GCF_014284475.1 | ['GH808_RS08595', 'GH808_RS03350'] | ['GH808_RS08600', 'GH808_RS10855'] | ['Y', 'Y'] | [['Y', 'N'], ['N', 'N']] | ['GH808_RS06185', 'GH808_RS06170'] | ['GH808_RS06185', 'GH808_RS06170'] | ['GH808_RS06180'] | ['GH808_RS06165'] |
| *Acetobacterium malicum* | GCA_014284495.1 | ['GH811_03365', 'GH811_03590', 'GH811_03750', 'GH811_03865', 'GH811_01580'] | ['GH811_03360', 'GH811_01420'] | ['Y', 'Y'] | [['Y', 'N'], ['N', 'N'], ['N', 'N'], ['N', 'N'], ['N', 'N']] | ['GH811_14760'] | ['GH811_14760'] | --- | --- |
| *Acetobacterium paludosum* | GCF_008086595.1 | ['FZC41_RS05030'] | ['FZC41_RS05025', 'FZC41_RS08725'] | ['Y', 'Y'] | [['Y', 'N']] | --- | --- | --- | --- |
| *Acetobacterium tundrae* | GCF_008086615.1 | ['FZC40_RS07445', 'FZC40_RS13050'] | ['FZC40_RS07440', 'FZC40_RS09950'] | ['Y', 'Y'] | [['Y', 'N'], ['N', 'N']] | --- | --- | --- | --- |
| *Acetobacterium wieringae* | GCF_008107585.1 | ['FXB42_RS13260', 'FXB42_RS01650'] | ['FXB42_RS13255', 'FXB42_RS01495'] | ['Y', 'Y'] | [['Y', 'N'], ['N', 'N']] | ['FXB42_RS10035'] | ['FXB42_RS10035'] | --- | --- |
| *Acetobacterium woodii* DSM 1030 | GCA_000247605.1 | ['Awo_c21740'] | ['Awo_c22060'] | ['Y'] | [['N']] | --- | --- | --- | --- |
| *Acetohalobium arabaticum* DSM 5501 | GCA_000144695.1 | ['Acear_0297'] | ['Acear_0533', 'Acear_0362'] | ['Y', 'Y'] | [['N', 'N']] | ['Acear_1098', 'Acear_1099', 'Acear_1097'] | ['Acear_1099', 'Acear_1097', 'Acear_1098'] | --- | --- |
| *Acetonema longum* DSM 6540 | GCF_000219125.1 | --- | --- | ['ND', '*'] | --- | ['ALO_RS00265', 'ALO_RS08220', 'ALO_RS08205', 'ALO_RS00270', 'ALO_RS00275'] | ['ALO_RS00275', 'ALO_RS08220', 'ALO_RS00270', 'ALO_RS08205', 'ALO_RS00265'] | ['ALO_RS00245', 'ALO_RS08215'] | ['ALO_RS00240', 'ALO_RS08200', 'ALO_RS09345'] |
| *Alkalibaculum bacchi* | GCF_003317055.1 | ['DES36_RS03130'] | ['DES36_RS00630'] | ['Y'] | [['N']] | --- | --- | --- | --- |
| *Blautia hydrogenotrophica* DSM 10507 | GCF_001404935.1 | --- | ['ARA85_RS00980'] | ['Y'] | --- | --- | --- | --- | --- |
| *Blautia schinkii* | GCF_013304825.1 | --- | ['HFM85_RS08490'] | ['Y'] | --- | --- | --- | --- | --- |
| *Calderihabitans maritimus* | GCF_002207765.1 | --- | --- | ['ND', '*'] | --- | ['KKC1_RS08925', 'KKC1_RS14735', 'KKC1_RS06640', 'KKC1_RS06640', 'KKC1_RS06640', 'KKC1_RS01160', 'KKC1_RS08930', 'KKC1_RS01175', 'KKC1_RS08935', 'KKC1_RS14740', 'KKC1_RS14745'] | ['KKC1_RS06640', 'KKC1_RS06640', 'KKC1_RS08935', 'KKC1_RS01175', 'KKC1_RS14735', 'KKC1_RS14745', 'KKC1_RS08930', 'KKC1_RS01160'] | ['KKC1_RS06645', 'KKC1_RS08905', 'KKC1_RS14720', 'KKC1_RS01165'] | ['KKC1_RS06665', 'KKC1_RS01180', 'KKC1_RS08900', 'KKC1_RS14710', 'KKC1_RS10630'] |
| *Carboxydothermus ferrireducens* DSM 11255 | GCF_000427565.1 | --- | --- | ['ND', '*'] | --- | ['CARFE_RS0112825', 'CARFE_RS0112830', 'CARFE_RS0112835'] | ['CARFE_RS0112835', 'CARFE_RS0112825', 'CARFE_RS0112830'] | ['CARFE_RS0112805'] | ['CARFE_RS0112800'] |
| *Carboxydothermus hydrogenoformans* | GCA_000012865.1 | --- | --- | ['ND', '*'] | --- | ['CHY_1417', 'CHY_1832', 'CHY_1832', 'CHY_1832', 'CHY_1416', 'CHY_1415'] | ['CHY_1415', 'CHY_1832', 'CHY_1832', 'CHY_1417', 'CHY_1416'] | ['CHY_1831', 'CHY_1421'] | ['CHY_1827', 'CHY_1422'] |
| *Carboxydothermus pertinax* | GCF_001950255.1 | --- | --- | ['ND', '*'] | --- | ['cpu_RS11170', 'cpu_RS01910', 'cpu_RS01910', 'cpu_RS01910', 'cpu_RS11165', 'cpu_RS11160'] | ['cpu_RS11160', 'cpu_RS11170', 'cpu_RS01910', 'cpu_RS01910'] | ['cpu_RS01915', 'cpu_RS11190'] | ['cpu_RS01935', 'cpu_RS11195'] |
| *Clostridium aceticum* | GCA_001042715.1 | ['CACET_c17430'] | ['CACET_c16320'] | ['Y'] | [['N']] | ['CACET_c33260', 'CACET_c33270', 'CACET_c33250', 'CACET_c29680'] | ['CACET_c33250', 'CACET_c33260', 'CACET_c33270', 'CACET_c29680'] | --- | --- |
| *Clostridium autoethanogenum* DSM 10061 | GCA_000484505.1 | ['CAETHG_3226'] | ['CAETHG_3227'] | ['Y'] | [['Y']] | --- | --- | --- | --- |
| *Clostridium carboxidivorans* | GCA_001038625.1 | ['Ccar_25730', 'Ccar_07835'] | ['Ccar_25735'] | ['Y'] | [['Y'], ['N']] | ['Ccar_06750', 'Ccar_06735'] | ['Ccar_06750', 'Ccar_06735'] | --- | ['Ccar_06755'] |
| *Clostridium coskatii* | GCA_001675205.1 | ['CLCOS_05690'] | ['CLCOS_05700'] | ['Y'] | [['Y']] | --- | --- | --- | --- |
| *Clostridium drakei* | GCA_003096175.1 | ['B9W14_04695', 'B9W14_12340'] | ['B9W14_04700'] | ['Y'] | [['Y'], ['N']] | ['B9W14_11370', 'B9W14_11385'] | ['B9W14_11385', 'B9W14_11370'] | --- | ['B9W14_11390'] |
| *Clostridium formicaceticum* | GCA_002080475.1 | ['CLFO_18400', 'CLFO_12270'] | ['CLFO_17180'] | ['Y'] | [['N'], ['N']] | ['CLFO_36480', 'CLFO_36490', 'CLFO_36470'] | ['CLFO_36470', 'CLFO_36480', 'CLFO_36490'] | --- | --- |
| *Clostridium kluyveri* DSM 555 | GCA_000016505.1 | ['CKL_1263', 'CKL_2767'] | ['CKL_1264'] | ['Y'] | [['Y'], ['N']] | --- | --- | --- | --- |
| *Clostridium ljungdahlii* DSM 13528 | GCA_000143685.1 | ['CLJU_c11350'] | ['CLJU_c11360'] | ['Y'] | [['Y']] | --- | --- | --- | --- |
| *Clostridium magnum* DSM 2767 | GCF_900129955.1 | ['BUC18_RS08450', 'BUC18_RS02475'] | ['BUC18_RS08445', 'BUC18_RS02470'] | ['Y', 'Y'] | [['Y', 'N'], ['N', 'Y']] | ['BUC18_RS02805', 'BUC18_RS02790'] | ['BUC18_RS02790'] | ['BUC18_RS02800'] | ['BUC18_RS02785'] |
| *Clostridium ragsdalei* P11 | GCF_001675165.1 | ['CLRAG_RS05955'] | ['CLRAG_RS05950'] | ['Y'] | [['Y']] | --- | --- | --- | --- |
| *Clostridium scatologenes* | GCA_000968375.1 | ['CSCA_2973', 'CSCA_1298'] | ['CSCA_2972'] | ['Y'] | [['Y'], ['N']] | ['CSCA_1581', 'CSCA_1578'] | ['CSCA_1578', 'CSCA_1581'] | --- | ['CSCA_1577'] |
| *Eubacterium aggregans* | GCF_900107815.1 | ['BLW33_RS05705'] | ['BLW33_RS04770'] | ['Y'] | [['N']] | --- | --- | --- | --- |
| *Eubacterium limosum* | GCA_000807675.2 | ['B2M23_08890'] | ['B2M23_19790'] | ['Y'] | [['N']] | --- | --- | --- | --- |
| *Marvinbryantia formatexigens* DSM 14469 | GCF_900102475.1 | --- | ['BLR58_RS17940'] | ['Y'] | --- | ['BLR58_RS16225', 'BLR58_RS16230', 'BLR58_RS16220', 'BLR58_RS16215'] | ['BLR58_RS16215', 'BLR58_RS16220', 'BLR58_RS16230'] | --- | --- |
| *Moorella mulderi* DSM 14980 | GCF_001594015.1 | --- | --- | ['ND', '*'] | --- | ['MOMUL_RS08150', 'MOMUL_RS08155', 'MOMUL_RS08160'] | ['MOMUL_RS08160', 'MOMUL_RS08155', 'MOMUL_RS08150'] | ['MOMUL_RS08130'] | ['MOMUL_RS08125'] |
| *Moorella thermoacetica* | GCF_001874605.1 | --- | --- | ['ND', '*'] | --- | ['MTJW_RS04625', 'MTJW_RS11345', 'MTJW_RS11365', 'MTJW_RS04630', 'MTJW_RS11350', 'MTJW_RS04635'] | ['MTJW_RS04635', 'MTJW_RS11365', 'MTJW_RS11350', 'MTJW_RS04630', 'MTJW_RS11345', 'MTJW_RS04625'] | ['MTJW_RS04605', 'MTJW_RS11360'] | ['MTJW_RS11340', 'MTJW_RS04600'] |
| *Oxobacter pfennigii* | GCF_001317355.1 | --- | ['OXPF_RS13305'] | ['Y'] | --- | ['OXPF_RS01035', 'OXPF_RS20260', 'OXPF_RS20250', 'OXPF_RS20255', 'OXPF_RS01030', 'OXPF_RS01025'] | ['OXPF_RS20260', 'OXPF_RS01025', 'OXPF_RS20255', 'OXPF_RS20250', 'OXPF_RS01035', 'OXPF_RS01030'] | ['OXPF_RS01055'] | ['OXPF_RS01060'] |
| *Rhodobacter capsulatus* SB 1003 | GCF_000021865.1 | --- | ['RCAP_RS16250'] | ['Y', '*'] | --- | ['RCAP_RS10560', 'RCAP_RS07605', 'RCAP_RS07610', 'RCAP_RS07615'] | ['RCAP_RS10560', 'RCAP_RS07610', 'RCAP_RS07615'] | ['RCAP_RS07575'] | ['RCAP_RS07530'] |
| *Sporomusa acidovorans* DSM 3132 | GCF_900101845.1 | ['BLR65_RS14630', 'BLR65_RS04290', 'BLR65_RS03790'] | ['BLR65_RS14625'] | ['Y'] | [['Y'], ['N'], ['N']] | ['BLR65_RS15875', 'BLR65_RS15880'] | --- | --- | --- |
| *Sporomusa malonica* | GCF_900176355.1 | ['B9A22_RS13135', 'B9A22_RS03705'] | ['B9A22_RS13130'] | ['Y'] | [['Y'], ['N']] | ['B9A22_RS12830', 'B9A22_RS12815'] | ['B9A22_RS12830', 'B9A22_RS12815'] | --- | ['B9A22_RS12810'] |
| *Sporomusa ovata* DSM 2662 | GCF_000445445.1 | ['SOV_RS03895'] | ['SOV_RS03900'] | ['Y'] | [['Y']] | ['SOV_RS04180', 'SOV_RS04195'] | --- | --- | ['SOV_RS04200'] |
| *Sporomusa silvacetica* DSM 10669 | GCF_002257705.1 | ['SPSIL_RS12000', 'SPSIL_RS09415'] | ['SPSIL_RS11995'] | ['Y'] | [['Y'], ['N']] | ['SPSIL_RS11675', 'SPSIL_RS11660'] | ['SPSIL_RS11675'] | --- | ['SPSIL_RS11655'] |
| *Sporomusa sphaeroides* DSM 2875 | GCF_900042765.1 | ['SSPH_RS15205'] | ['SSPH_RS15210'] | ['Y'] | [['Y']] | ['SSPH_RS02485', 'SSPH_RS02470'] | ['SSPH_RS02470'] | --- | ['SSPH_RS02465'] |
| *Sporomusa termitida* | GCA_007641255.1 | ['SPTER_14240'] | ['SPTER_14250'] | ['Y'] | [['Y']] | ['SPTER_39500', 'SPTER_39470'] | ['SPTER_39470'] | --- | ['SPTER_39460'] |
| *Terrisporobacter mayombei* | GCF_014333445.1 | --- | ['H9L25_RS04125'] | ['Y'] | --- | --- | --- | --- | --- |
| *Thermacetogenium phaeum* DSM 12270 | GCA_000305935.1 | --- | --- | ['ND', '*'] | --- | ['Tph_c26280', 'Tph_c21360', 'Tph_c26310'] | ['Tph_c26280', 'Tph_c26310', 'Tph_c21360'] | ['Tph_c26290', 'Tph_c21350'] | ['Tph_c26330', 'Tph_c21320'] |
| *Thermoanaerobacter kivui* | GCA_000763575.1 | --- | --- | ['ND', '*'] | --- | ['TKV_c19720', 'TKV_c01230', 'TKV_c19710'] | ['TKV_c19710', 'TKV_c01230', 'TKV_c19720'] | ['TKV_c19690', 'TKV_c01240'] | ['TKV_c19740', 'TKV_c01310'] |
| *Treponema primitia* ZAS-2 | GCF_000214375.1 | --- | ['TREPR_RS17540', 'TREPR_RS10980'] | ['Y', 'Y'] | --- | --- | --- | --- | --- |

**^a^** All acetogens with available full genome sequences were selected from Table 2 in Bengelsdorf et al. (2018).

**^b^** Potential *rseC* genes were screened in each genome by protein sequence comparison to the RseC of *C. ljungdahlii* (CLJU_C11350).

**^c^** The presence of (potential) RNF-gene clusters was defined by potential RnfC and RnfD protein sequence within +/- 500 bp of each other. The RnfC (CLJU_c11360) and RnfD (CLJU_c11370) protein sequences of *C. ljungdahlii* were used as reference sequences.

**^d^** Potential *rseC* genes were screened for being located +/- 500 bp of the identified *rnfC* gene.

**^e^** The presence of (potential) Ech-gene clusters was screened using the protein sequences of Ech2A1, Ech2A2, Ech2B, EchE2*,* from *T. kivui* (TKV_c19720, TKV_c19710, TKV_c19690, TKV_C19740) as references.
* Indicates that a potential G subunit of the RNF-gene cluster is missing (other potential subunits ABCDE were found).

Y, yes

N, no

ND, not detected

**References**

Bayat, H., Modarressi, M.H., and Rahimpour, A. (2018). The conspicuity of CRISPR-Cpf1 system as a significant breakthrough in genome editing. *Current Microbiology* 75(1)**,** 107-115. doi: 10.1007/s00284-017-1406-8.

Bengelsdorf, F.R., Beck, M.H., Erz, C., Hoffmeister, S., Karl, M.M., Riegler, P., et al. (2018). Bacterial Anaerobic Synthesis Gas (Syngas) and CO_2_ + H_2_ Fermentation. *Advances in Applied Microbiology* 103**,** 143-221. doi: 10.1016/bs.aambs.2018.01.002.

Biegel, E., Schmidt, S., Gonzalez, J.M., and Müller, V. (2011). Biochemistry, evolution and physiological function of the Rnf complex, a novel ion-motive electron transport complex in prokaryotes. *Cellular and Molecular Life Sciences* 68(4)**,** 613-634. doi: 10.1007/s00018-010-0555-8.

De Las Peñas, A., Connolly, L., and Gross, C.A. (1997). The σ^E^‐mediated response to extracytoplasmic stress in *Escherichia coli* is transduced by RseA and RseB, two negative regulators of σ^E^. *Molecular Microbiology* 24(2)**,** 373-385. doi: 10.1046/j.1365-2958.1997.3611718.x

Ding, H., and Demple, B. (1997). *In vivo* kinetics of a redox-regulated transcriptional switch. *Proceedings of the National Academy of Sciences of the United States of America* 94(16)**,** 8445-8449. doi: 10.1073/pnas.94.16.8445.

Dong, H., Tao, W., Zhang, Y., and Li, Y. (2012). Development of an anhydrotetracycline-inducible gene expression system for solvent-producing *Clostridium acetobutylicum*: A useful tool for strain engineering. *Metabolic Engineering* 14(1)**,** 59-67. doi: 10.1016/j.ymben.2011.10.004.

Drake, H.L., Gossner, A.S., and Daniel, S.L. (2008). Old acetogens, new light. *Annals of the New York Academy of Sciences* 1125**,** 100-128. doi: 10.1196/annals.1419.016.

Emerson, D.F., Woolston, B.M., Liu, N., Donnelly, M., Currie, D.H., and Stephanopoulos, G. (2019). Enhancing hydrogen-dependent growth of and carbon dioxide fixation by *Clostridium ljungdahlii* through nitrate supplementation. *Biotechnology and Bioengineering* 116(2)**,** 294-306. doi: 10.1002/bit.26847.

Fagerlund, R.D., Staals, R.H., and Fineran, P.C. (2015). The Cpf1 CRISPR-Cas protein expands genome-editing tools. *Genome Biology* 16**,** 251. doi: 10.1186/s13059-015-0824-9.

Heap, J.T., Pennington, O.J., Cartman, S.T., and Minton, N.P. (2009). A modular system for *Clostridium* shuttle plasmids. *Journal of Microbiological Methods* 78(1)**,** 79-85. doi: 10.1016/j.mimet.2009.05.004.

Huang, H., Chai, C., Li, N., Rowe, P., Minton, N.P., Yang, S., et al. (2016). CRISPR/Cas9-based efficient genome editing in *Clostridium ljungdahlii*, an autotrophic gas-fermenting bacterium. *ACS Synthetic Biology* 5(12)**,** 1355-1361. doi: 10.1021/acssynbio.6b00044.

Koo, M.S., Lee, J.H., Rah, S.Y., Yeo, W.S., Lee, J.W., Lee, K.L., et al. (2003). A reducing system of the superoxide sensor SoxR in *Escherichia coli*. *The EMBO Journal* 22(11)**,** 2614-2622. doi: 10.1093/emboj/cdg252

Livak, K.J., and Schmittgen, T.D. (2001). Analysis of relative gene expression data using real-time quantitative PCR and the 2^−ΔΔCT^ method. *Methods* 25(4)**,** 402-408. doi: 10.1006/meth.2001.1262.

Missiakas, D., Mayer, M.P., Lemaire, M., Georgopoulos, C., and Raina, S. (1997). Modulation of the *Escherichia coli* σ^E^ (RpoE) heat‐shock transcription‐factor activity by the RseA, RseB and RseC proteins. *Molecular Microbiology* 24(2)**,** 355-371. doi: 10.1046/j.1365-2958.1997.3601713.x.

Möller, S., Croning, M.D., and Apweiler, R. (2001). Evaluation of methods for the prediction of membrane spanning regions. *Bioinformatics* 17(7)**,** 646-653.

Woolston, B.M., Emerson, D.F., Currie, D.H., and Stephanopoulos, G. (2018). Rediverting carbon flux in *Clostridium ljungdahlii* using CRISPR Interference (CRISPRi). *Metabolic Engineering*. doi: 10.1016/j.ymben.2018.06.006.

Zetsche, B., Gootenberg, J.S., Abudayyeh, O.O., Slaymaker, I.M., Makarova, K.S., Essletzbichler, P., et al. (2015). Cpf1 is a single RNA-guided endonuclease of a class 2 CRISPR-Cas system. *Cell* 163(3)**,** 759-771. doi: 10.1016/j.cell.2015.09.038.

Zhao, R., Liu, Y., Zhang, H., Chai, C., Wang, J., Jiang, W., et al. (2019). CRISPR-Cas12a-mediated gene deletion and regulation in *Clostridium ljungdahlii* and its application in carbon flux redirection in synthesis gas fermentation. *ACS Synthetic Biology* 8(10)**,** 2270-2279. doi: 10.1021/acssynbio.9b00033.
